# Effect of dulaglutide on functional and structural MRI changes during cigarette smoking cessation

**DOI:** 10.1093/braincomms/fcag410

**Published:** 2026-10-27

**Authors:** Clara O Sailer, Sophia Lengsfeld, David Coynel, Davide Zanchi, Nica Jeanloz, Fabienne Baur, Cemile Bathelt, Katja Bologna, Tanja Vukajlovic, Sandrine A Urwyler, Mirjam Christ-Crain, Bettina Winzeler

**Affiliations:** Department of Endocrinology, Diabetology and Metabolism, University Hospital Basel, 4031 Basel, Switzerland; Department of Clinical Research, University Hospital Basel, University of Basel, 4031 Basel, Switzerland; Division of Endocrinology, Diabetes, and Metabolism, Mass General Brigham, Harvard Medical School, Boston, MA 02115, USA; Department of Endocrinology, Diabetology and Metabolism, University Hospital Basel, 4031 Basel, Switzerland; Department of Clinical Research, University Hospital Basel, University of Basel, 4031 Basel, Switzerland; Division of Cognitive Neuroscience, Department of Biomedicine, University of Basel, 4055 Basel, Switzerland; Research Cluster Molecular and Cognitive Neurosciences, Department of Biomedicine, University of Basel, 4055 Basel, Switzerland; F. Hoffmann- La Roche, Roche Innovation Centre Basel, 4070 Basel, Switzerland; Stanford University Graduate School of Business, Stanford, CA 94305, USA; Department of Endocrinology, Diabetology and Metabolism, University Hospital Basel, 4031 Basel, Switzerland; Department of Clinical Research, University Hospital Basel, University of Basel, 4031 Basel, Switzerland; Department of Endocrinology, Diabetology and Metabolism, University Hospital Basel, 4031 Basel, Switzerland; Department of Clinical Research, University Hospital Basel, University of Basel, 4031 Basel, Switzerland; Department of Endocrinology, Diabetology and Metabolism, University Hospital Basel, 4031 Basel, Switzerland; Department of Clinical Research, University Hospital Basel, University of Basel, 4031 Basel, Switzerland; Department of Endocrinology, Diabetology and Metabolism, University Hospital Basel, 4031 Basel, Switzerland; Department of Clinical Research, University Hospital Basel, University of Basel, 4031 Basel, Switzerland; Department of Endocrinology, Diabetology and Metabolism, University Hospital Basel, 4031 Basel, Switzerland; Department of Clinical Research, University Hospital Basel, University of Basel, 4031 Basel, Switzerland; Department of Endocrinology, Diabetology and Metabolism, University Hospital Basel, 4031 Basel, Switzerland; Department of Clinical Research, University Hospital Basel, University of Basel, 4031 Basel, Switzerland; Department of Endocrinology, Diabetology and Metabolism, University Hospital Basel, 4031 Basel, Switzerland; Department of Clinical Research, University Hospital Basel, University of Basel, 4031 Basel, Switzerland; Department of Clinical Research, University Hospital Basel, University of Basel, 4031 Basel, Switzerland; Department of Endocrinology, Diabetology and Clinical Nutrition, University Hospital Zurich, 8091 Zurich, Switzerland

**Keywords:** GLP-1 receptor agonists, craving for cigarettes, nicotine addiction, reward, varenicline

## Abstract

Glucagon-like peptide-1 (GLP-1) receptor agonists are investigated for treating addiction. However, it remains unclear whether these agents modulate addiction-related neural circuitry. To address this question, we conducted a predefined neuroimaging subtrial of a larger randomized placebo-controlled trial. While the parent trial enrolled 255 participants with at least moderate nicotine dependence, this mechanistic study included a subset of 71 participants. Participants were randomized to receive either 1.5 mg dulaglutide (*N* = 35) or placebo (*N* = 36) for 12 weeks, both administered alongside a standard-of-care regimen of varenicline and behavioural counselling. Structural and functional MRI assessments were performed at baseline and after 12 weeks. Main outcome measures included abstinence rates, changes in craving for cigarette smoking, working memory performance, whole-brain functional activation when exposed to smoking and neutral videos, resting state functional connectivity, and grey matter volume changes from baseline to 12 weeks. We found that 12 weeks of intervention (59 participants completed the 12-week visit (*N* = 57 usable functional, *N* = 58 usable structural data)), abstinence rates were comparable between the dulaglutide (54%) and placebo (68%) groups (*P* = 0.27). Across the entire cohort, craving for cigarette smoking significantly decreased (*P* < 0.001) and was consistently higher during smoking-cue versus neutral-cue videos (*P* < 0.001). Working memory improved significantly among participants who achieved smoking abstinence (*P* = 0.02). While we observed small-to-medium reductions in addiction- and reward-related brain activation from baseline to week 12 across the entire cohort, no significant differences were found between dulaglutide and placebo for any clinical, functional, or structural outcomes. Our findings demonstrate that dulaglutide conferred no incremental benefit over a potent standard-of-care regimen of varenicline and behavioural counselling. These findings are critical for the field, as they suggest a potential ceiling effect of current first-line therapies and define the boundaries of GLP-1 receptor agonists in addiction. While dulaglutide did not modulate cue-induced neural activation in this adjunctive context, this study establishes the feasibility of using longitudinal mechanistic neuroimaging to guide the development of future incretin-based interventions.

## Teaser

Sailer *et al*. report findings from a neuroimaging subtrial investigating the glucagon-like peptide-1 receptor agonist dulaglutide for smoking cessation. They demonstrate that adding dulaglutide to standard-of-care smoking cessation treatment with varenicline and behavioural counselling provides no additional clinical benefit for abstinence rates, nor functional or structural addiction-related brain regions.

## Graphical Abstract

**Graphical Abstract:**
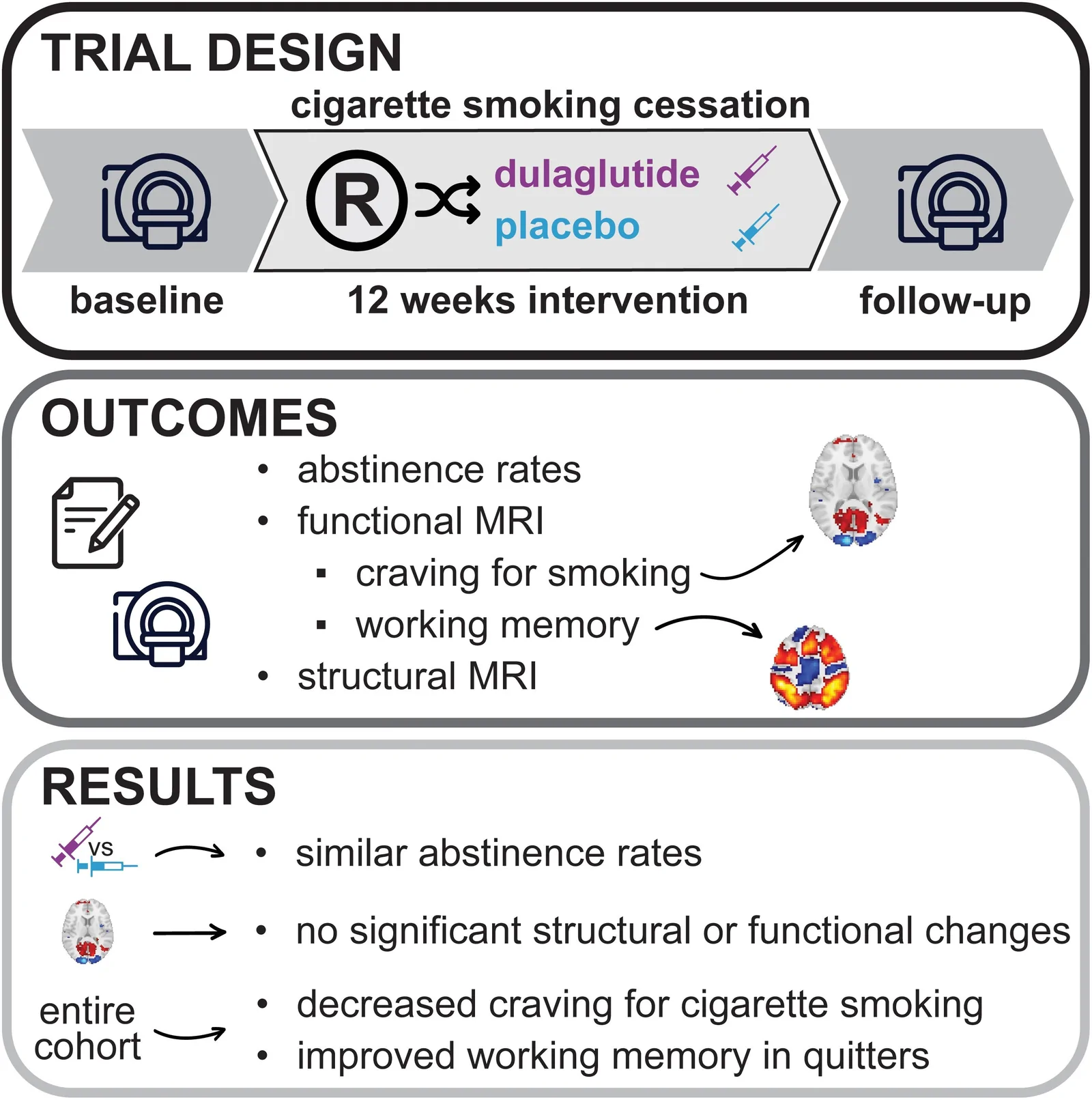

## Introduction

Cigarette smoking remains a leading cause of preventable mortality globally.^1-3^ Despite the implementation of comprehensive strategies combining behavioural interventions with pharmacotherapy, like varenicline, bupropion, and nicotine replacement therapy, long-term abstinence rates among individuals who aim to quit smoking remain low.^4,5^ To develop more effective interventions, research should focus on understanding the neurobiological underpinnings of cigarette smoking and of smoking cessation treatments.

Functional neuroimaging has identified key circuits involved in nicotine dependence, including those involved in reward regulation [nucleus accumbens (NAcc), ventral tegmental area (VTA)], emotion processing (amygdala, insula), and executive control [anterior cingulate cortex (ACC), dorsolateral prefrontal cortex (dlPFC)].^6-9^ The standard-of-care smoking cessation treatment varenicline (a partial agonist of neuronal nicotinic acetylcholine receptors) modulates activation in these circuits. Specifically, varenicline reduces task-based cue-reactivity in the amygdala and thalamus while enhancing resting-state functional connectivity between executive control regions, effectively strengthening cognitive regulation over incentive salience.^10-12^ While chronic cigarette smoking is associated with grey matter volume loss, specifically in the frontal lobes, the insula, and the thalamus,^13-16^ 12 weeks of varenicline treatment in 73 individuals who smoke cigarettes has been shown to attenuate grey matter volume loss in the orbitofrontal cortex and thalamus, suggesting that even short-term pharmacological intervention can induce measurable structural neuroplasticity.^17^

Glucagon-like-peptide 1 (GLP-1) receptor agonists (RAs), medications known for their glucose- and weight-lowering effects, have emerged as promising candidates for treating addictive behaviours.^18-24^ GLP-1 receptors are highly expressed in mesolimbic reward system (NAcc, VTA), where they modulate dopaminergic signalling, which is crucial in addiction and reward behaviours,^25,26^ as well as in the lateral septum and nucleus of the solitary tract (NTS), where they regulate endogenous eating behaviour and exert weight-lowering effects.^27,28^ The reinforcing effects of nicotine are primarily driven by a dopaminergic surge within the mesolimbic reward system, specifically the projection from the ventral tegmental area (VTA) to the nucleus accumbens (NAcc). GLP-1 receptors are expressed within these regions, and their activation has been shown to attenuate drug-induced dopamine release.^29^ Consequently, GLP-1 RAs are hypothesized to ‘de-value’ nicotine by diminishing its reinforcing properties, a mechanism supported by preclinical models showing reduced self-administration.^20^ Consistently, a preliminary randomized placebo-controlled trial in humans investigating exenatide found increased cigarette smoking abstinence rates and reduced craving for cigarette smoking after six weeks compared to placebo.^30^ In contrast, we previously demonstrated, in a double-blind randomized placebo-controlled trial with 255 participants, no benefit of a 12-week treatment with the GLP-1 RA dulaglutide in terms of abstinence rates or craving but less weight gain associated with cigarette smoking cessation.^31^

The aim of this predefined, neuroimaging subtrial was to investigate the neural mechanisms, or lack thereof, by which dulaglutide, compared to placebo, modulates behaviour and addiction-related neural circuits during smoking cessation. We hypothesized that 12-week dulaglutide treatment, compared to placebo, would (i) improve behavioural outcomes (increase abstinence rates, reduce craving, and improve working memory performance), (ii) reduce cue-induced activation in addiction- and reward-related regions and alter resting-state functional connectivity and cerebral blood flow, and (iii) attenuate grey matter volume loss in cortical and subcortical regions. As exploratory hypothesis, we further investigated these changes between participants who quit compared to those who continued smoking cigarettes.

## Materials and methods

### Trial design and participants

The previously reported single-centre, randomized, double-blind, placebo-controlled trial included 255 participants and investigated the GLP-1 RA dulaglutide, compared to placebo, on cigarette smoking cessation and weight change.^31-33^ The present neuroimaging trial included a subset of 71 participants and was conducted at the University Hospital Basel, Switzerland, from 2017 to 2022. Eligible participants, were between 18 and 60 years old, were included if they smoked ≥10 cigarettes per day, exhibited at least moderate nicotine dependence (defined by a Fagerstroem Score of ≥5 points, see Supplementary Methods), or were currently smoking <10 cigarettes per day and had a tobacco-related disease such as pulmonary, cardiovascular, cerebrovascular, gastrointestinal disease, or cancer. Participants had to be willing to quit smoking. Regardless of the study, all participants received standard-of-care treatment with varenicline and behavioural counselling.^34^ Exclusion criteria were pregnancy (including wish to become pregnant within the next 3 months) or breastfeeding, pre-existing treatment with GLP-1 RAs, history of pancreatitis, severe renal insufficiency (eGFR <30 ml/min/1.73 m^2^), unstable psychiatric condition, anorexia nervosa, medical conditions affecting the brain (e.g. stroke, epilepsy, space-occupying lesions, multiple sclerosis, Parkinson’s disease, dementia, transient ischaemic attack), current use of medications that alter brain function (e.g. antidepressant, antipsychotic, antiepileptic medication), current illicit drug use including cannabis, alcohol (≤1 drink per day), claustrophobia, cardiac pacemaker, electronic device, or ferromagnetic metal foreign bodies.^32^

Written informed consent was obtained from all participants after explanation of all trial procedures and before inclusion. The trial was conducted according to the Declaration of Helsinki and Good Clinical Practice guidelines, registered at clinicaltrials.gov (NCT03204396), approved by the Northwestern and Central Switzerland Ethics Committee, and the national agency for the authorization and supervision of therapeutic products (Swissmedic, Berne, Switzerland).

### Trial procedures

The trial was conducted in addition to standard-of-care smoking cessation consisting of daily varenicline treatment, initiated at study inclusion and titration per tolerance with goal varenicline of 2 mg/day, and behavioural counselling focusing on smoking cessation strategies according to patients’ needs (Supplementary Methods).^31^ The procedures and timeline for the present trial are schematically depicted in Supplementary Fig. 1.

At baseline and after the 12-week intervention, clinical measures including weight and smoking urge (assessed by the German version of the Questionnaire on Smoking Urge, QSU-G, see Supplementary Methods) were collected. Cigarette smoking status was evaluated through self-report, end-expiratory exhaled carbon monoxide (Micro+™ Smokerlyzer®, ≤10 ppm for participants who quit), and semi-quantitative urine cotinine level in spot urine (Urine Cotinine All Test COT 3 in 1, negative for participants who quit).^35,36^ The trial intervention was administered weekly through subcutaneous injections (dulaglutide [Trulicity®]: 0.75 mg for the first and 1.5 mg for the consecutive visits, or placebo: 0.5 ml of natrium chloride 0.9% [Supplementary Methods]).

Neuroimaging sessions were conducted at baseline (before administration of the first trial intervention) and after the 12-week intervention (time frame ranging from 10 to 12 weeks). To standardize craving for smoking, participants were required to abstain from smoking for a minimum of two hours before undergoing the neuroimaging visit. Cigarette smoking status was confirmed through self-report and end-expiratory exhaled carbon monoxide (≤10 ppm for participants who quit).

Behavioural measures included abstinence rates, craving for smoking, and working memory performance (assessed by the n-back task) (Supplementary Methods).^37^ During the craving for smoking task, participants were presented with smoking and neutral videos in a pseudo-randomized fashion.^38,39^ Craving for smoking was assessed using a visual analogue scale (VAS, 1 = no craving for smoking, 7 = maximal craving for smoking).

Neuronal activation during functional MRI were assessed using the blood-oxygen-level-dependent signal during the craving for smoking task, the n-back task, and resting-state.^37,38^ The resting-state scan took place with dimmed light, and participants were lying with their eyes open. Changes in cerebral blood flow (perfusion) by means of arterial spin labelling (ASL) were also collected.

For structural measures, grey matter brain volumes of cortical and subcortical regions were extracted from the T1-weighted sequences. During the structural scans, participants were instructed to lie quietly without performing a task or falling asleep.

Details on all MRI procedures are provided in the Supplementary Methods.

### Choice of study medication

When the study was designed and approved by the local institutional review board in Switzerland, dulaglutide and exenatide were the only approved weekly GLP-1 RAs. Dulaglutide was chosen because of the more rapid achievement of therapeutic (steady state) plasma concentrations compared to exenatide.

### Blinding

Injection devices of dulaglutide and placebo were not identical. Therefore, injections were performed by unblinded study staff otherwise not involved in the trial. Participants wore blindfolds during the drug injection and could not see the injection site or the injection device. Participants, study physician, study nurses, and other individuals involved in the study conduct were blinded to treatment allocation.^27,28,32^

### Neuroimaging data processing

Neuroimaging data were acquired on a 3T Siemens Prisma scanner at the University Hospital of Basel, Switzerland. The neuroimaging data were pre-processed on a modality-specific basis. Calculations were performed at the Center for Scientific Computing of the University of Basel (sciCORE, http://scicore.unibas.ch/). Pre-processing was performed using fMRIPrep v20.2.6 (https://fmriprep.org/) for the task-based and resting-state fMRI data, where the main pre-processing steps consisted in coregistration to the structural image, spatial normalization, unwarping, and noise component extraction. The resting-state data were further analysed with xcpEngine (https://xcpengine.readthedocs.io/). FreeSurfer v7.2.0 was used for pre-processing the T1-weighted images, and BASIL vFSL/6.0.2 (https://asl-docs.readthedocs.io/) was used for the ASL data. A complete description of the different pipelines is provided in the Supplementary Methods.

### Statistical analyses

#### Sample size and analysis sets

The neuroimaging trial was a predefined substudy designed to generate hypotheses regarding the neuroimaging effects of GLP-1 RA in cigarette smoking cessation. We expected that a minimum of 60 participants would allow for meaningful results to generate new hypotheses.^32^

The baseline data set included all randomized participants with usable baseline neuroimaging data. For the MRI follow-up data set a follow-up MRI and at least 80% adherence to the trial medication (=10/12 doses) were required, see Fig. 1.

**Figure 1:**
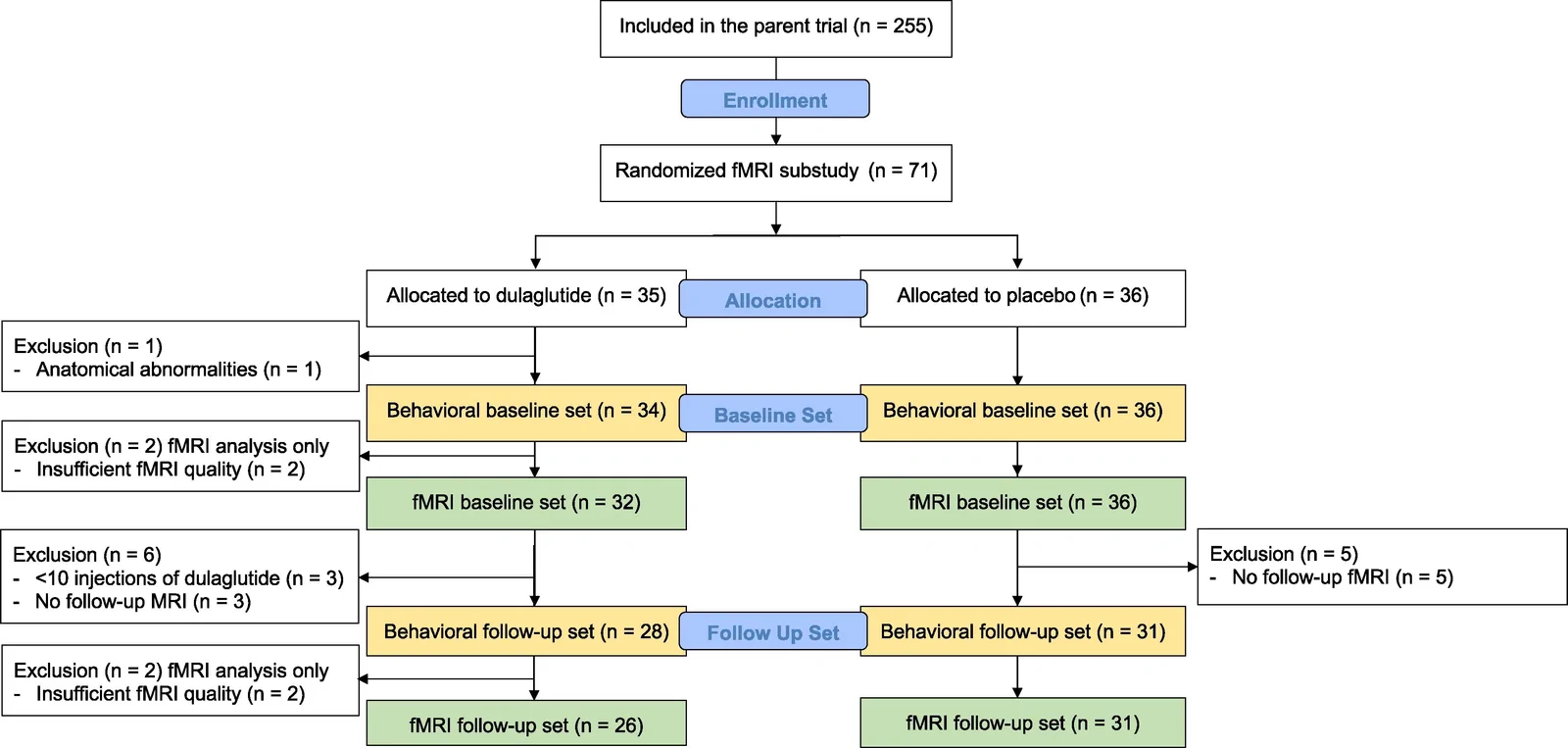
**Consort flow diagram**. The baseline data set included all randomized participants of the neuroimaging trial who had usable baseline neuroimaging data. The follow-up set included all participants with usable neuroimaging data at baseline and after 12 weeks and a trial intervention adherence rate of at least 80% in the dulaglutide group. Functional and structural neuroimaging data were deemed to be usable when no quality control problems or substantial anatomical abnormalities were identified during pre-processing. The functional and structural neuroimaging data sets slightly differed in their respective quality control pipelines. Abbreviation: fMRI = functional magnetic resonance imaging, *n* = number, MRI = magnetic resonance imaging.

Differences in outcomes between the two groups were analysed, and frequentist *P*-values provided for each outcome of each comparison. Summary statistics were computed, including reporting of frequencies of nominal and ordinal data and distribution parameters of numerical data at baseline and 12-week visit.

#### Behavioural data

To test whether dulaglutide compared to placebo increases abstinence rates or reduces urge for smoking (QSU-G), we used a Wilcoxon Rank Sum Test. To test whether dulaglutide compared to placebo reduces craving for cigarette smoking, we computed a generalized linear mixed-effects model with Poisson distribution to account for VAS count data (1–7). Intervention (dulaglutide versus placebo), timepoint (baseline versus 12-week visit), and stimulus (smoking versus neutral videos) were set as fixed effects and participants as random effect.

To evaluate whether dulaglutide compared to placebo improves working memory performance, we computed a linear mixed effect model with intervention (dulaglutide versus placebo), n-back (0- versus 1-/2-back), and session (baseline versus 12-week visit) as fixed effects and participants as random effect.

To evaluate the above hypothesis as exploratory analyses between participants who quit compared to those who continued cigarette smoking, we repeated the models with smoking status (those who quit versus continued smoking) instead of intervention (dulaglutide versus placebo), pooling both treatment arms across the entire cohort.

#### Functional neuroimaging data

To assess that, at baseline, smoking versus neutral videos show increased activation within addiction and reward related key brain regions, we compared activation during the smoking task for the contrast smoking versus neutral videos at baseline in all patients, both at the whole-brain level and in 28 pre-defined ROIs relevant for addiction and reward processes. The regions stem from the Hill-Bowen-2021 meta-analysis (Supplementary Table 1, Supplementary Fig. 2), to which the medial orbitofrontal cortex was added based on its role in reward processing and nicotine dependence.^8,10,11^ These analyses were repeated to assess differences in activation from baseline to the 12-week visit for the smoking versus neutral video contrast.

To evaluate whether dulaglutide compared to placebo leads to reduced activation within addiction and reward related key brain regions after the 12-week intervention, we compared activation during the smoking task between participants receiving dulaglutide versus placebo at the whole-brain level and within the above-mentioned 28 ROIs. To explore whether those who quit smoking show reduced activation, we repeated the above-mentioned analyses based on smoking status.

For the working memory task, brain activation was compared between the 2-back blocks and 0-back blocks.

Whole-brain *P*-values were corrected for multiple comparisons using the familywise error procedure (FWE, two-sided *P* < 0.05), while *P*-values for the ROI-based analyses were corrected for multiple comparisons using the false discovery rate (FDR) procedure.

For both resting-state functional connectivity and cerebral blood flow (ASL), we report baseline characteristics as well as the difference from baseline to the 12-week visit for the entire cohort, as well as based on intervention group, and smoking status. *P*-values for those analyses were corrected for multiple comparisons using the FDR procedure.

All functional neuroimaging group-level analyses included age, craving for cigarette smoking, n-back accuracy, pre-MRI carbon monoxide, pack years, Fagerstroem score as covariates. Further details regarding the statistical analyses of the neuroimaging data can be found in the Supplementary Statistical Analyses section.

#### Structural data

To explore the hypothesis that dulaglutide compared to placebo leads to attenuated grey matter volume loss in cortical and subcortical grey matter volumes, we computed regression models adjusted for sex and estimated total intracranial volume from baseline to the 12-week visit between participants receiving dulaglutide and placebo. To explore the hypothesis that cigarette smoking cessation leads to attenuated grey matter volume loss in cortical and subcortical brain regions, we computed above-mentioned regression models between those who quit versus continued smoking. We used the Desikan-Killiany Atlas to delineate the temporal, frontal, parietal, and occipital lobes from the extracted volumes.^40^ Subcortical ROIs were analysed, i.e. insula, amygdala, hippocampus, thalamus, cerebellum cortex, caudate and pallidum. *P*-value were corrected for multiple comparisons using the FDR.

## Results

### Baseline characteristics

Between February 2018 and July 2021, a total of 71 participants were enrolled and randomized to dulaglutide (*N* = 35) or placebo (*N* = 36). One participant was excluded because of posttraumatic anatomical changes, thus 70 were considered for the baseline data set. 59 completed the 12-week neuroimaging visit, of whom 57 had usable functional and 58 usable structural data, see Fig. 1 and Supplementary Methods. Participants were predominantly female (*N* = 41, 58.6%) with a mean age of 37.8 years [standard deviation (SD) 10.9], a mean body mass index (BMI) of 26.2 kg/m^2^ (SD 4.1) and a normal range median haemoglobin A1c (HbA1c) of 5.3% [interquartile range (IQR) 5, 5.5]. Median lifetime smoking exposure was 13.5 pack years (IQR 9, 22.8) and participants smoked 20 cigarettes per day (IQR 15, 20) at time of study inclusion. Mean Fagerstroem score was 6.9 points (SD 1.2), and total sum QSU-G score was 116.1 points (38.8), consistent with moderate to severe nicotine dependence. See Table 1 for all baseline characteristics and Supplementary Table 2 for baseline characteristics by sex.

**Table 1:** Baseline characteristics

	Overall	Dulaglutide	Placebo
Number of participants	*N* = 70	*N* = 34	*N* = 36
Age (years), mean (SD)	37.8 (10.9)	36.0 (11.4)	39.5 (10.3)
Sex, *n* (%)			
male female	29 (41) 41 (59)	16 (44) 18 (56)	13 (38) 23 (62)
Weight (kg), mean (SD)	79.8 (17.2)	78.5 (16.1)	81.1 (18.5)
BMI (kg/m^2^), mean (SD)	26.2 (4.1)	25.7 (4.1)	26.7 (4.2)
HbA1c (%), median [IQR]	5.3 [5.0, 5.5]	5.4 [5.2, 5.6]	5.2 [5.0, 5.5]
Caucasian, *n* (%)	68 (97)	34 (94)	34 (100)
Handedness, *n* (%)			
right left	67 (94) 3 (4)	34 (94) 2 (6)	33 (97) 1 (3)
Pack years, median [IQR]	13.5 [9.0, 22.8]	18.0 [10.0, 26.0]	11.5 [8.0, 17.0]
Cigarettes per day, median [IQR]	20.0 [15.0, 20.0]	20.0 [15.0, 20.0]	18.0 [15.0, 20.0]
End-expiratory exhaled carbon monoxide (ppm), median [IQR]	16.0 [10.8, 21.2]	19.0 [13.0, 22.0]	15.0 [10.0, 19.5]
Fagerstroem total sum score, mean (SD)	6.8 (1.2)	7.0 (1.4)	6.6 (0.9)
Craving for smoking QSU-G (total sum score), mean (SD)	116.1 (38.8)	119.6 (42.2)	112.4 (35.1)

Abbreviations: BMI = Body mass index, HbA1c = haemoglobin A1c, IQR = interquartile range, QSU-G = Questionnaire on Smoking urges - German version, SD = Standard deviation, *n* = number, VAS = Visual Analogue Scale.

### Behavioural data

In line with the main trial, cigarette smoking abstinence rates were high following the 12-week intervention: abstinence rate in the dulaglutide group was 15 (54%) and in the placebo group 21 (68%) (*P* = 0.27). Median time to smoking cessation was 14 days (IQR 10, 22).

At week 12, most participants reported adherence to varenicline, with 86% in the dulaglutide, 84% in the placebo group. QSU-G scores more than halved following the 12-week intervention, without a between-group difference for intervention (estimated mean difference −9.10 (95% CI −22.26, 3.95), *P* = 0.18) (Supplementary Table 3).

Baseline craving scores during the craving for smoking task were high and comparable based on interventions (dulaglutide rate ratio 0.98 (95% CI 0.86, 1.13), 0.81, effect size −0.02 (95% CI −0.15, 0.12)). Higher craving scores were reported during smoking versus neutral videos [smoking videos estimated difference 1.97 (95% CI 1.86, 2.09), <0.001, effect size 0.68 (95% CI 0.62, 0.73)] (Fig. 2, Supplementary Table 4).

**Figure 2:**
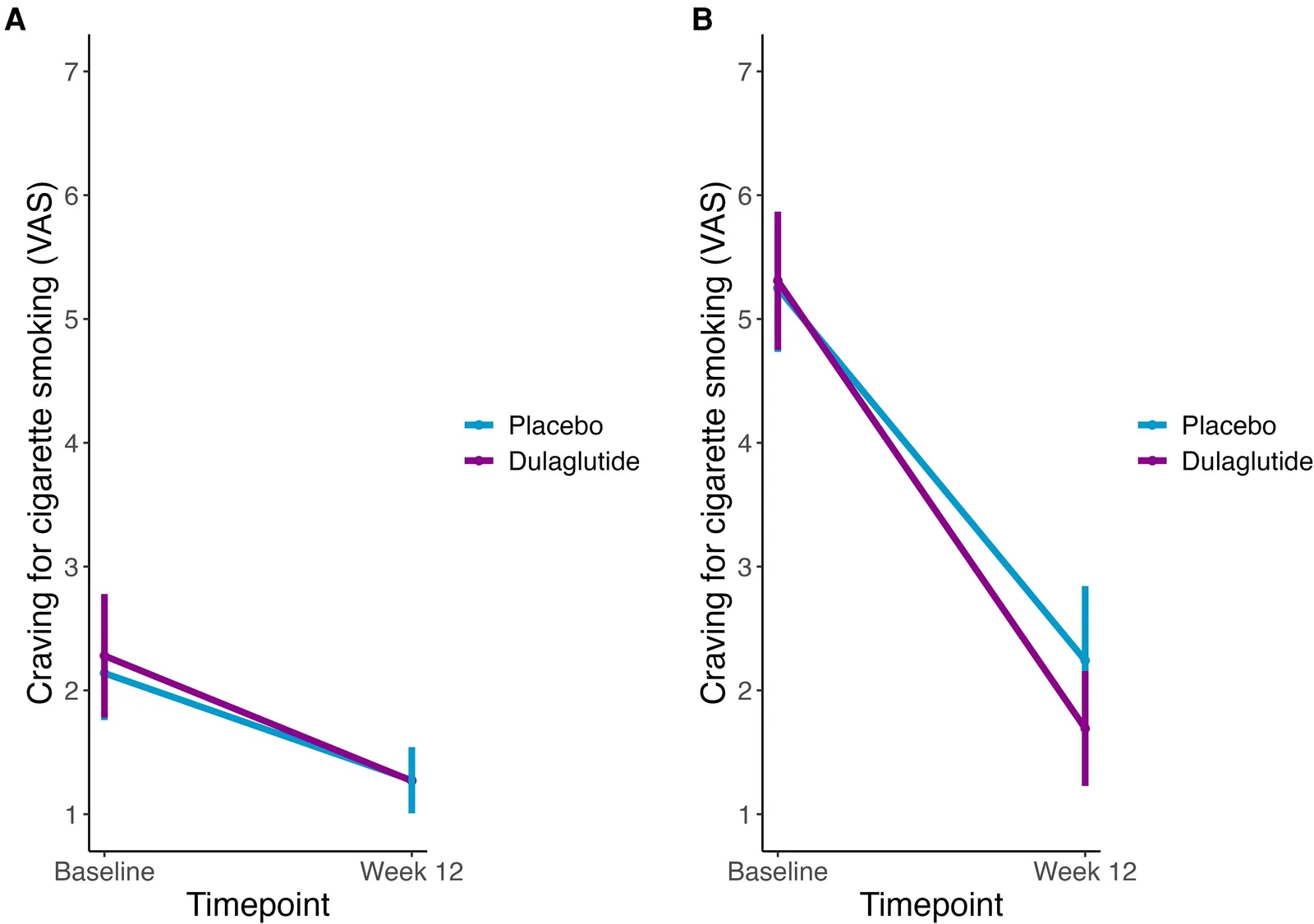
**Changes in craving for cigarette smoking from baseline to the 12-week visit based on intervention.** (**A**) Neutral stimulus. (**B**) Smoking stimulus. Mean craving for cigarette smoking scores (Visual Analogue Scale, VAS) at baseline and Week 12 for placebo (blue line) and dulaglutide (purple line). Data points represent sample mean and error bars indicate the 95% confidence intervals (CI). Total *N* = 59, Dulaglutide = 26, Placebo = 31.

Following the 12-week intervention, overall craving scores were lower compared to baseline in both the dulaglutide and placebo group (dulaglutide rate ratio 0.48 (95% CI 0.45, 0.50) *P* < 0.001, effect size −0.37 (95% CI −0.39, −0.34)), especially when exposed to smoking videos (rate ratio 1.85 (95% CI 1.76, 1.94) *P* < 0.001, effect size 0.61 (95% CI 0.56, 0.66)). However, there was no difference based on intervention (rate ratio 0.94 (95% CI 0.814, 1.08) *P* = 0.35, effect size −0.07 (95% CI −0.20, 0.07)) or smoking status (rate ratio 1.11 (95% CI 0.97, 1.28) *P* = 0.12, effect size 0.11 (95% CI −0.03, 0.24)), (Fig. 2, Supplementary Fig. 3, Supplementary Tables 4, 5).

Following the 12-week intervention, there was no difference in the working memory performance task between dulaglutide and placebo (reaction time: dulaglutide estimated difference −19.0 (95% CI −51.33, 13.52); *P* = 0.25, accuracy: dulaglutide estimated difference 0.003 (95% CI −0.005, 0.012), *P* = 0.46) (Supplementary Table 6). Participants who continued smoking cigarettes had a slower reaction time than those who quit (participants who continued smoking: estimated difference 40.49 (95% CI 8.67, 72.36), *P* = 0.02) (Supplementary Table 7).

### Functional neuroimaging data

#### Craving for cigarette smoking paradigm

##### Whole-brain analyses at baseline: all participants

Across the entire cohort, whole-brain voxel-wise significant clusters of increased activation when viewing smoking videos were found in the medial orbitofrontal cortex, bilateral hippocampus and parahippocampal gyrus, bilateral lingual gyrus, bilateral intracalcarine cortex, bilateral cuneus, and precuneus/posterior cingulate cortex, see Fig. 3A and Table 2. Whole-brain, voxel-wise significant clusters showing an increased activation when viewing neutral videos were found in the bilateral precentral and postcentral cortex, bilateral superior parietal lobule, bilateral lateral occipital cortex, bilateral occipital pole, left superior posterior cerebellum and bilateral inferior posterior cerebellum, see Fig. 3A and Table 2. The majority of those regions had at least a small to medium effect size (Cohen’s |*d*| > 0.3).

**Figure 3:**
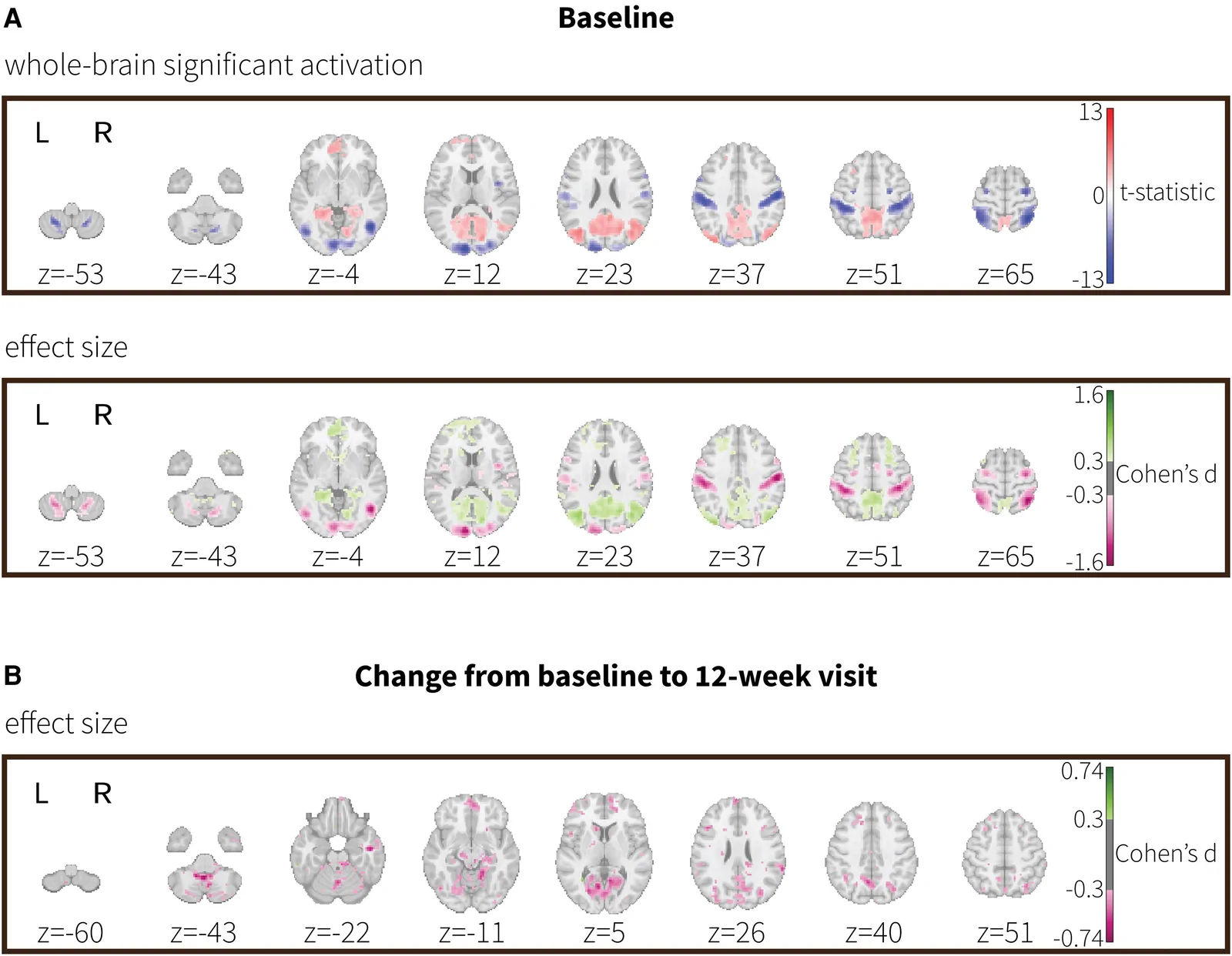
**Craving for cigarette smoking task: brain activation for the smoking versus neutral videos contrast (A) at baseline and (B) difference between baseline and the 12-week visit, in the entire cohort**. (**A**) The top row represents the whole-brain corrected statistical brain map of the one-sample *t*-test (*N* = 66). Red colours represent voxels with significantly more activation for smoking versus neutral videos, while blue colours represent significantly more activation for neutral versus smoking videos. The images are corrected for multiple comparisons using familywise error procedure (FWE) (*P*-corrected <0.05). The bottom row shows the corresponding Cohen’s d effect size map. Green colours represent a greater effect for smoking versus neutral videos, while purple colours represent a greater effect for neutral versus smoking videos. Only values of |*d*| > 0.3 are represented. (**B**) Cohen’s *d* effect size map (*N* = 55). Green colours represent an increase in activation from baseline to week 12, while purple colours represent a decrease in activation. Only values of |*d*| > 0.3 are represented. *Z* depicts the axial slice's coordinate in MNI (Montreal Neurological Institute) space. *L* indicates left hemisphere, and *R* indicates right hemisphere.

**Table 2:** Brain activations for the smoking task at baseline and 12-week visit

Contrast	Region	T-value	*P*-value	X	Y	Z
**Baseline**						
*smoking versus neutral*	Left lateral occipital cortex	7.36	0.0013	−36.5	−69.5	26.8
	Right lateral occipital cortex	7.31	0.0009	38.5	−75.5	26.8
	Right intracalcarine cortex	7.11	0.0002	17.5	−81.5	5.74
	Right lingual gyrus	6.19	0.0013	20.5	−42.5	−11.8
	Right cuneal cortex	6.02	0.0002	23.5	−66.5	23.3
	Left frontal medial cortex	5.53	0.0076	−12.5	47.5	−8.3
	Left lingual gyrus	5.5	0.0003	−21.5	−45.5	−4.79
*neutral versus smoking*	Right superior parietal lobule	−13.1	0.0001	32.5	−51.5	65.4
	Right lateral occipital cortex	−11.8	0.0001	47.5	−66.5	−4.79
	Left occipital pole	−11.3	0.0001	−9.5	−96.5	12.8
	Left postcentral gyrus	−11.2	0.0001	−48.5	−27.5	40.8
	Right precentral gyrus	−10.6	0.0002	29.5	−15.5	61.9
	Left lateral occipital cortex	−9.19	0.0001	−45.5	−75.5	2.23
	Left precentral gyrus	−8.56	0.0042	−27.5	−15.5	58.4
	Left cerebellum	−8.31	0.0028	−27.5	−57.5	−53.9
	Right cerebellum	−7.75	0.0077	23.5	−57.5	−50.4
	Right precentral gyrus	−7.06	0.0085	59.5	8.5	33.8
	Left precentral gyrus	−6.61	0.0234	−60.5	5.5	37.3
**12-week visit**						
*smoking versus neutral*	Left lateral occipital cortex	6.63	0.0304	−51.5	−81.5	26.8
	Right lateral occipital cortex	5.63	0.0318	47.5	−66.5	19.8
*neutral versus smoking*	Right postencentral gyrus	10.2	0.0002	59.5	−15.5	33.8
	Left lateral occipital cortex	9.86	0.0002	−48.5	−72.5	−4.79
	Right lateral occipital cortex	9.13	0.0004	47.5	−60.5	−4.79
	Left supramarginal gyrus	8.77	0.0002	−54.5	−27.5	40.8
	Left cerebellum	8.49	0.0074	−27.5	−57.5	−53.9
	Left occipital pole	8.26	0.0002	−6.5	−99.5	5.74
	Right cerebellum	6.56	0.0199	20.5	−60.5	−53.9

Peak voxels of the clusters showing a whole-brain significant effect for the smoking versus neutral videos and neutral versus smoking videos contrasts: corresponding T-values, *P*-values and coordinates in MNI space (X, Y, Z).

##### Whole-brain analyses at follow-up: all participants

There were no significant whole-brain differences in activation across the entire cohort from baseline to the 12-week visit for the smoking versus neutral videos, but the effect size map showed small to medium reduction in activation for regions that were more active in the smoking versus neutral videos contrast (Fig. 3B).

##### Whole-brain analyses: group differences

We found no whole-brain differences in activation for the smoking versus neutral videos following the 12-week intervention, neither based on intervention group nor smoking status. Considering craving scores or n-back performance as additional covariates did not change the results.

##### Regions of interest analyses

When focusing on activation within the 28 predefined ROIs, we also did not find any significant difference in activation for the smoking versus neutral videos from baseline to the 12-week visit (Supplementary Table 8).

#### Working memory task

At baseline and across the entire cohort, the 2-back blocks compared to 0-back blocks elicited significant and greater whole-brain clusters of activations in fronto-parietal and cerebellar regions (Supplementary Fig. 4). Specifically, the bilateral superior parietal gyrus, bilateral superior and middle frontal gyrus, bilateral frontal pole, the bilateral thalamus, and anterior insula. Regions more active during the 0-back blocks were the frontal medial cortex, posterior cingulate gyrus and bilateral central opercular cortex. The majority of those regions had at least a small to medium effect size (|*d*| > 0.3).

There were no whole-brain significant differences in activation between baseline and the 12-week visit in the entire cohort. The effect size map shows a mixed pattern of small clusters with small to medium increase or reduction in activation (Supplementary Fig. 5). There were no whole-brain significant differences in activation after 12 weeks based on intervention or smoking status.

#### Resting state functional connectivity and arterial spin labelling

We found no significant changes in resting-state whole-brain connectivity and perfusion between sessions overall, nor according to intervention or smoking status (Supplementary Figs 6 and 7).

### Structural neuroimaging data

There were no differences in grey matter volume in the bilateral lobar (frontal, parietal, temporal, occipital lobe) or subcortical (insula, thalamus, caudate, putamen, pallidum, hippocampus, amygdala) regions at baseline or the 12-week visit between interventions or smoking status, see Supplementary Tables 9 and 10. In a regression model that included age and total intracranial volume as covariates, assessing volume changes from baseline to the 12-week visit, we found no difference based on either intervention or smoking status, see Supplementary Tables 11 and 12.

## Discussion

We report that adjunctive dulaglutide treatment did not significantly modulate the clinical or neurobiological trajectories of cigarette smoking cessation when compared to placebo. While the standard-of-care regimen (varenicline and behavioural counselling) successfully reduced craving for cigarette smoking after 12 weeks and led to a small- to medium-effect-size reduction of cue-elicited activation in reward-related circuits, dulaglutide conferred no incremental benefit across any primary or secondary endpoint. Specifically, analyses revealed no significant between-group differences in abstinence rates, working memory reaction times, whole-brain functional connectivity, reactivity to smoking cues, resting-state connectivity, or grey matter volume changes over 12 weeks. These data provide mechanistic limitations of GLP-1 RA in the context of intensive, current first-line cigarette smoking cessation therapies.

As expected, a noteworthy reduction in overall craving for cigarette smoking was observed during smoking cessation, while participants received adjunctive treatment with dulaglutide or placebo in addition to standard of care with varenicline and behavioural counselling. These results align with previous studies indicating that interventions combining varenicline and behavioural counselling effectively diminish the craving to smoke.^10^

Accuracy in working memory performance has been shown to be reduced in individuals who chronically smoke cigarettes.^41^ We found faster reaction time in those who achieved abstinence compared to those who continued smoking. While this may suggest a modest improvement in cognitive processing speed following cigarette smoking cessation, we found no functional neuroimaging correlates. Therefore, these results should be interpreted cautiously; while they may indicate a trend towards improved cognitive efficiency, they do not definitively establish a restoration of complex ‘working performance’ or broader functional recovery within this 12-week timeframe.^41^

In line with the literature, at baseline, when all participants were smoking and treatment-naïve, smoking cues elicited robust activation in addiction and reward related brain regions, confirming the craving task’s validity for activating craving-related brain circuitries.^38,39,42^ We demonstrate that smoking videos evoked greater activation than neutral videos in reward- and addiction-related brain regions (e.g. medial orbitofrontal cortex, bilateral hippocampus, bilateral intracalcarine cortex, precuneus/posterior cingulate cortex). This pattern is frequently observed in cue-reactivity paradigms and may reflect the engagement of the default mode network during salient stimuli or the processing of neutral social information. The presence of these distinct patterns across different contrasts supports the reliability of the task in differentiating between salient addiction cues and neutral environmental stimuli.

The small- to medium-effect-sized reductions of activation in reward regions after 12 weeks likely reflect the potent influence of varenicline and behavioural counselling. Varenicline consistently attenuates cue-induced activation in the dorsolateral prefrontal cortex, amygdala, thalamus, and striatal regions, as well as reduces craving-related neural responses.^12,43,44^ Our findings support this effect of varenicline and behavioural counselling on smoking cessation but demonstrates no additional benefit from dulaglutide. This is consistent with the results of our main trial, where abstinence rates were unexpectedly high across the entire cohort ranging up to 68%. These null findings are scientifically significant as they likely reflect a ‘ceiling effect’; the high efficacy of the standard-of-care regimen may have masked any incremental pharmacological signal from dulaglutide. One mechanistic explanation of this ceiling effect is the neurobiological overlap of varenicline and GLP-1 RAs. Both agents modulate dopamine signalling within the mesolimbic reward system, specifically the VTA, and NAcc.^27,28^ From a mechanistic standpoint, it would therefore be informative to study GLP-1 RAs against an alternative option that does not act on this same circuitry, for example, nicotine replacement therapy or placebo. Indeed, an earlier randomized clinical pilot trial found that adding exenatide to nicotine replacement therapy facilitated cessation and reduced craving.^30^

Our results contrast with pilot trials of other GLP-1 RAs, like exenatide, which report reduced craving and withdrawal.^30^ However, central GLP-1 RA effects appear to vary across agents and individual metabolic phenotypes. For instance, higher BMI may potentiate GLP-1-mediated reductions in reward responsivity.^45-48^ In our cohort of predominantly normal-weight individuals, dulaglutide may have provided insufficient central modulation to influence nicotine-cue reactivity. Furthermore, divergent neuropharmacokinetics among GLP-1 RAs suggests that agents with greater central nervous system bioavailability may be required to achieve clinical effects in addiction settings.^49,50^

Strengths of this trial include the prospective, double-blind, placebo-controlled design in motivated individuals attempting cigarette smoking cessation, and longitudinal neuroimaging assessment during a validated craving paradigm. Few studies have examined GLP-1 signalling in nicotine addiction with mechanistic imaging, and this work contributes early evidence on dulaglutide’s neural effects in humans.

Some limitations should be considered. Concomitant varenicline and behavioural counselling likely contributed to the strong cessation outcomes and may have obscured any incremental effect of dulaglutide. In addition, dulaglutide dose, duration, and central nervous system penetrance may have been insufficient, and our predominantly normal-weight sample may not reflect metabolic phenotypes in which GLP-1–based modulation of reward circuitry is more pronounced.

In conclusion, while our smoking-cessation programme led to a marked clinical and neural improvements, dulaglutide did not provide any additional benefits over placebo. Publishing these null findings is critical to inform the field about the boundaries of GLP-1 RA-based intervention in the context of high-intensity standard of care. Future research should aim to disentangle the specific neural and clinical contributions of pharmacotherapy versus behavioural therapy and further clarify the neurobiological role of incretin-based treatments within smoking-cessation strategies.

## Supplementary Material

fcag410_Supplementary_Data

## Data Availability

We share de-identified, individual participant-level data that underlie the results reported in this article and related documents, including the study protocol and the statistical analysis plan. Data will be available with the publication of our manuscript and receipt of a request detailing the study hypothesis and statistical analysis plan. All requests should be sent do the corresponding author. Code used to generate the results can be found here: https://osf.io/4hnxr/.

## Trial registration

NCT03204396.
