# Supplementary material for: Effect of dulaglutide on functional and structural MRI changes during cigarette smoking cessation
Source: Brain Commun. 2026 Oct 27;8(6):fcag410. doi: 10.1093/braincomms/fcag410 (PMC13748752; doi:10.1093/braincomms/fcag410)
Supplement: Supplementary file 1 [file fcag410_supplementary_data.pdf]

Supplementary Material

Effect of dulaglutide on functional and structural MRI changes during cigarette smoking cessation

Clara O. Sailer* 1,2,3; Sophia Lengsfeld* 1,2; David Coynel* 4,5, Davide Zanchi 6,7; Nica Jeanloz, 1,2; Fabienne Baur, 1,2; Cemile Bathelt 1,2; Katja Bologna 1,2; Tanja Vukajlovic 1,2; Sandrine A. Urwyler 1,2, Mirjam Christ-Crain 1,2; and Bettina Winzeler 2,8

* Clara O. Sailer, Sophia Lengsfeld, and David Coynel contributed equally to this work.

1 Department of Endocrinology, Diabetology and Metabolism, University Hospital Basel, Basel, Switzerland

2 Department of Clinical Research, University Hospital Basel, University of Basel, Basel, Switzerland

3 Division of Endocrinology, Diabetes, and Metabolism, Mass General Brigham, Harvard Medical School, Boston, Massachusetts, USA

4 Division of Cognitive Neuroscience, Department of Biomedicine, University of Basel, Basel, Switzerland

5 Research Cluster Molecular and Cognitive Neurosciences, Basel, Switzerland

6 F. Hoffmann- La Roche, Roche Innovation Centre Basel, Basel, Switzerland

7 Stanford University Graduate School of Business, Stanford, California, USA

8 Department of Endocrinology, Diabetology and Clinical Nutrition, University Hospital Zurich, Zurich, Switzerland

Correspondence:

Bettina Winzeler, MD

Department of Endocrinology, Diabetology and Clinical Nutrition, Raemistrasse 100, 8091 Zurich, Switzerland

Phone: 41.432533404; Email: Bettina.winzeler@usz.ch

ORCID: 0000-0001-8305-2700

Table of content

Methods 4
Fagerstroem score for cigarette dependence 4
German version of the questionnaire on cigarette smoking urges (QSU-G) 4
Standard cigarette smoking cessation care 4
Blinding 4
Behavioral tasks 4
Craving for cigarette smoking task 4
Working memory performance task 5
Magnetic resonance imaging (MRI) procedures 5
Statistical analyses 6
Craving for cigarette smoking regions 6
Task based functional MRI 6
Resting-state 7
Arterial spin labelling 7
Structural MRI 8
MRI boilerplate 8
Tables 11
Supplementary Table 1: Coordinates of the regions of interest for the cigarette smoking task analysis. 11
Supplementary Table 2: Baseline characteristics for women and men separately. 12
Supplementary Table 3: QSU-G total sum scores based on intervention at baseline and the 12-week visit. 12
Supplementary Table 4: Craving for cigarette smoking (VAS) during functional MRI based on intervention at baseline and the 12-week visit. 13
Supplementary Table 5: Craving for cigarette smoking (VAS) during functional MRI based on cigarette smoking status at baseline and the 12-week visit. 14
Supplementary Table 6: N-Back task based on intervention at the 12-week visit. 15
Supplementary Table 7: N-Back task based on cigarette smoking status at the 12-week visit. 15
Supplementary Table 8: Regions of interest analysis for the cigarette smoking task. 16
Supplementary Table 9: Gray matter brain volume based on intervention at baseline and the 12-week visit. 19
Supplementary Table 10: Gray matter brain volume according to cigarette smoking status at baseline and the 12-week visit. 20
Supplementary Table 11: Regression model for cortical and subcortical grey matter brain volume based on intervention at baseline and the 12-week visit. 21
Supplementary Table 12: Regression model for cortical and subcortical grey matter brain volume based on cigarette smoking status at baseline and the 12-week visit. 22
Figures 23
Supplementary Figure 1: Trial procedures and timeline. 23
Supplementary Figure 2: Regions of interest for the cigarette smoking task. 24

Supplementary Figure 3: Changes in craving for cigarette smoking from baseline to the 12-week visit based on cigarette smoking status. 30
Supplementary Figure 4: 2-back versus 0-back blocks at baseline, in the whole population. 30
Supplementary Figure 5: Difference in brain activation for the 2-back versus 0-back blocks between baseline and the 12-week visit, in the whole population. 31
Supplementary Figure 6: Resting-state functional connectivity, in the whole population... 32
Supplementary Figure 7: Average perfusion values per region. 33

Methods

Fagerstroem score for cigarette dependence

The Fagerstroem score for nicotine dependence is a standardized instrument designed to assess the severity of nicotine addiction.[1] It contains six items that evaluate the quantity of cigarette consumption, the compulsion to use, and nicotine dependence. In scoring the Fagerstroem score, yes/no items are scored with 0 or 1 and multiple-choice items are scored from 0 to 3. The items are summed up to yield a total score of 0 to 10. The higher the total Fagerstroem score, the more intense the participant's dependence to nicotine. Scores of 0 to 2 imply a low, 3 to 4 a moderate, 5 to 6 a high and 7 to 10 a very high nicotine dependence.

German version of the questionnaire on cigarette smoking urges (QSU-G)

The German version of the questionnaire on cigarette smoking urges (QSU-G) is a 32-item self-report questionnaire concerning desire and intention to smoke, with perceived as rewarding (factor 1) and desire to smoke in anticipation of relief from negative (factor 2), respectively.[2] Dis- and agreement is measured using a Likert-type scale ranging from 1=strongly disagree to 7=strongly agree; questions 4, 6, 8, 10, 11, 16, 17, 21, 22, 26, 27, 28, 32 are measured reversed (1=7, 2=6, 3=5 etc). 32-224 points can be achieved; higher scores imply higher cigarette smoking urge.

Standard cigarette smoking cessation care

The standard of care included behavioral counselling and treatment with the nicotinic partial receptor agonist varenicline according to national guidelines.[3,4] Titration regimen of varenicline and details of the behavior counselling were as followed: Varenicline titration regimen: days 1-3: 0.5mg/day, days 4-7: 2x0.5mg/day, day 8 to week 12: 2x1mg/day. Behavioral counselling was done by certified cigarette smoking cessation advisors. All participants attended a first face to face meeting and received information about available cigarette smoking cessation strategies. The follow-up appointments were arranged according to participants' needs.

Blinding

As injection devices of dulaglutide and placebo were not identical, the trial drug injection was performed by otherwise not involved unblinded study staff from the Clinical Trial Unit (CTU) while the blinded study staff belonged to the Department of Endocrinology, Diabetology and Metabolism, University Hospital Basel. Before start of the study, a specific standard operating procedure (SOP) was developed by the CTU and all unblinded staff were trained according to this SOP. To ensure blinding of the participants, they were blindfolded during the drug injection.

Behavioral tasks

Craving for cigarette smoking task

The craving for cigarette smoking task developed by Brody et al. consisted of an on-off block-design with two active conditions: cigarette smoking videos and neutral control videos.[5,6] Each participant completed two runs at each session, featuring a pseudo-randomized sequence of five cigarette smoking and five control videos. Each video had a duration of 45 seconds and was followed by a Visual Analogue Scale (VAS) for 2.5 seconds (1=no craving, 7=maximal craving). The curser was initial set in the middle of the scale and participants were asked to move the cursor to indicate their craving for cigarette smoking. A rest period consisting of a fixation cross was displayed following the VAS for 10 seconds. Before the scanning session,

participants were provided with instructions on the procedure and familiarized with the task through a training run outside of the MRI scanner.

Working memory performance task

Working memory performance was assessed using the well-established n-back task. The task consists of a sequence of letters presented one at a time in the center of a screen.[7] Each stimulus was presented for 1 second followed by a blank screen for 2 seconds. During the 0-back condition, participants were required to press the button with the right hand when the letter „X" appeared. During 1-back and 2-back conditions, participants were instructed to press the button if the currently presented letter was the same as the prior one (1-back condition) or two trials beforehand (2-back condition). The three conditions were presented in ten alternating 30 second blocks (5×0-back, 2×1-back, and 3×2-back) matched for the number of target letters per block (i.e., 2 or 3), in a pseudo-random order.

Magnetic resonance imaging (MRI) procedures

Eligible participants for functional and structural neuroimaging analyses

Two participants were excluded from the functional MRI (fMRI) dataset as their T1-weighted images were not properly segmented in the fMRIprep pipeline. A participant's fMRI run (task-based or resting-state) was excluded from further analysis if it met one of those criteria: 1) mean framewise displacement (FD) was greater than 0.5mm; 2) more than 20% of time points exceeded an FD of 0.5mm; 3) FD at any time point was greater than 5mm. One participant was excluded from the structural analyses as it had grey matter brain volumes that were considered outlier in more than 10 areas.

Field map

For each session, a B0 field map was acquired with the following parameters: dual-echo gradient-recall echo sequence, field of view 224mm, matrix size 64*64, slice thickness 3.5mm, spacing between slices 4.025mm, 35 axial slices, echo time 1 4.92ms, echo time 2 7.38ms, repetition time 0.5s, flip angle 50º, voxel size 3.5mm3.

Task-based functional MRI

Functional neuronal changes were assessed through the surrogate of blood oxygenated level dependent (BOLD) signal, an indirect measure of neuronal activation. Whole-brain single-echo EPI (echo-planar imaging) sequences were acquired with the following parameters: field of view 228mm, matrix size 76*76, no parallel imaging, slice thickness 3mm, spacing between slices 3.51mm, 40 axial slices, volume coverage in Z direction 140mm, interleaved acquisition, echo time 28ms, repetition time 2.5s, flip angle 82º, voxel size 3mm3.

The craving for cigarette smoking task consisted of 2 runs for each session, each comprising 245 volumes. The working memory task consisted of one run for each session, comprising 126 volumes.

Resting-state functional MRI

During the resting-state scans (5 minutes per session), participants were instructed to think of nothing specific and to not fall asleep. Each acquisition consisted in 168 volumes of whole-brain single-echo EPI sequences acquired with the following parameters: field of view 224mm, matrix size 64*64, GRAPPA (GeneRalized Autocalibrating Partial Parallel Acquisition) parallel imaging with an acceleration factor of 2, slice thickness 3.5mm, spacing between slices

4.025mm, 35 axial slices, volume coverage in Z direction 140.9mm, interleaved acquisition, echo time 28ms, repetition time (TR) 1.8s, flip angle 82º, voxel size 3.5*3.5*4.025 mm3.

Arterial spin labeling

A pulsed arterial spin labeling (ASL) sequence was acquired at each session. The sequence was the default 2D Siemens PICORE Q2T, with the following parameters: echo time 12ms, repetition time 3000ms, voxel size 4*4*4 mm3, bolus duration 700ms, and inversion time 1800ms. It consisted of one calibration M0 scan, followed by 45label/control pairs of scans. Participants were instructed to lie in the scanner without performing any task and lying as still as possible.

Statistical analyses

Craving for cigarette smoking regions

We used two different approaches to assess craving for cigarette smoking at baseline. Based on Figure 3 from the main manuscript, we identified brain regions showing a significant activation when participants viewed smoking videos at baseline, as compared to neutral videos. These brain regions can be hypothesized to represent neurobiological processes related to smoking craving and reward. We then investigated brain activation within these regions at follow-up, using a small-volume correction approach.

We also considered 28 ROIs representing craving/addiction/reward-related regions, consisting of the 27 ROIs from the Hill-Bowen meta-analysis as well as a ROI representing the medial orbitofrontal cortex. We investigated the difference in activation in those regions between baseline and follow-up, independently of treatment group or smoking status.

Task based functional MRI

The task-based fMRI data were pre-processed using *fMRIPrep* 20.2.6 [8,9] (RRID:SCR_016216), which is based on *Nipype* 1.7.0 [10,11] (RRID:SCR_002502). A complete description of the pre-processing pipeline is given below (section 3. MRI boilerplate).

Subject-level activation maps were computed using fitlins 0.9.2 (https://github.com/poldracklab/fitlins), based on the fMRIPrep-preprocessed dataset. Data were smoothed with an isotropic kernel of 6mm full width at half maximum.

For the craving for cigarette smoking task, each run and session, the following task trials were modeled: cigarette smoking video, neutral video, fixation cross, and VAS presentation. These trials had a duration of 45s, 45s, 4s and 10s, respectively. These regressors were convolved with the canonical haemodynamic response function (HRF), following SPM's model (https://www.fil.ion.ucl.ac.uk/spm/software/spm12/). A cosine drift model was implemented. The following movement-related regressors were included: translation and rotation along the [x, y, z] axes, their squared values, their first derivative and corresponding squared values. The contrast of interest was comparing cigarette smoking versus neutral videos. Both runs were combined by averaging the corresponding contrast estimates. In the case where only one run passed the quality control for excessive movement, only this one was used.

For the working memory performance (n-back task), the following task trials were modeled: 0-back blocks, 1-back blocks, and 2-back blocks. These regressors were convolved with the canonical HRF, following SPM's model. A cosine drift model was implemented. The following movement-related regressors were included: translation and rotation along the [x, y, z] axes, their squared values, their first derivative and corresponding squared values. The contrast of interest was comparing 2-back versus 0-back blocks.

For both task-based functional MRI analyses, baseline versus 12-week visit contrasts were constructed by subtracting 12-week visit from baseline estimated values.

Group-level analyses were conducted using non-parametric permutation tests implemented in Permutation Analysis of Linear Models (PALM, https://github.com/andersonwinkler/PALM, downloaded 2022-01-06). Ten thousand permutations were computed, errors being treated as being exchangeable, independent, and symmetric ("-ise -ee" option). We used the threshold-free cluster-enhancement (TFCE) approach to control for familywise error (FWE) at the cluster level. Threshold-free cluster-enhancement enhances cluster-like structures in the data without having to define somewhat arbitrary cluster thresholds beforehand. All t-tests were two-tailed ("-twotail" option), and the results were thresholded at a whole-brain corrected threshold level of $p<0.05$. The following analyses were conducted for all contrasts of interest: 1) baseline and 12-week visit mean activation, independently of group and cigarette smoking status; 2) baseline versus 12-week visit differences independently of group and cigarette smoking status, and separately based on intervention (dulaglutide vs placebo), and cigarette smoking status (persistent smokers vs quitters). The following covariates were included in all models: age, baseline carbon monoxide, pack years, and Fagerstroem score. For participants with missing data, their values were replaced by the group's mean. Additionally, we also report the corresponding effect sizes measured with Cohen's d.

We furthermore conducted a region of interest (ROI) based analysis, following Hill-Bowen et al.[12] Twenty-seven ROI were defined as 10mm diameter spheres around the peaks reported in Supplementary Table 1 of Hill-Bowen. We also included a ROI covering the medial orbitofrontal cortex, based on its role in reward processing and nicotine dependence. All 28 ROIs are depicted in Supplementary Figure 2. Mean contrast estimates were extracted for each ROI. For every region, we computed the difference in activation between 12-week visit and baseline for the cigarette smoking vs neutral videos contrast. The statistical models used for the whole-brain analyses were repeated with this set of ROIs.

Resting-state

The resting-state fMRI data were also pre-processed using *fMRIPrep* 20.2.6 (see the complete description below in section 3. MRI boilerplate). The resulting normalized BOLD time series were further processed with xcpEngine v1.2.4.[13] We applied a pre-defined functional connectivity pipeline (https://github.com/PennLINC/xcpEngine/blob/master/designs/fc-aroma.dsn), based on the ICA-AROMA approach.[14] The main objective of this pipeline is to remove motion artifacts and physiological signals from the BOLD time series in order to avoid spurious correlations when estimating functional connectivity. For a whole-brain functional connectivity approach, we were interested in the node-to-node functional connectivity outputs. Nodes were defined based on the 264-node Power atlas.[15] For each participant, node-to-node functional connectivity was quantified as the correlation between each node's processed time series. This resulted in 34'716 connectivity measures per participant. Group-level analyses were conducted for each connectivity value to determine whether resting-state connectivity significantly changed from baseline to the 12-week visit 1) in the whole population; 2) based on intervention (dulaglutide vs placebo) and cigarette smoking status (quitters vs persistent smokers). We used linear mixed-effects models as implemented in the lmer function of the lme4 R-package (R version 4.1.2).[16] Participants were defined as random effects, and the following covariates were included: age, baseline carbon monoxide, pack years, and Fagerstroem score. A false discovery rate (FDR)-correction for multiple comparisons was applied for every analysis.

Arterial spin labelling

ASL data were analyzed using the BASIL (Bayesian Inference for Arterial Spin Labeling) tool from FSL (FMRIB Software Library) (v6.0.2).[17] BASIL analysis included motion correction to the calibration image. Label and control images were subtracted, and a kinetic model was fitted

with modeling of the macrovascular component. The M0 calibration image acquired without ASL preparation or background suppression was used to quantify cerebral blood flow (CBF) in the cerebral spinal fluid (CSF) for calibration. The resulting calibrated perfusion image was further transformed to the Montreal Neurological Institute (MNI) space and used to extract region-specific average perfusion measurements. The brain was divided in 48 cortical and 21 subcortical ROIs following the Harvard-Oxford structural atlases.[18–21] Regions where participants had missing values due to brain coverage were excluded from further analyses (12 cortical regions: Inferior Temporal Gyrus anterior division, Inferior Temporal Gyrus posterior division, Inferior Temporal Gyrus temporooccipital part, Parahippocampal Gyrus anterior division, Temporal Fusiform Cortex anterior division, Temporal Fusiform Cortex posterior division, Cuneal Cortex, Intracalcarine Cortex, Lingual Gyrus, Occipital Fusiform Gyrus, Supracalcarine Cortex, Temporal Occipital Fusiform Cortex). 36 cortical and 12 subcortical regions were considered for further analyses. Regions where participants had a perfusion value outside of the group's average ±3 standard deviations were excluded from downstream analyses. Differences in perfusion between follow-up and baseline were computed by subtracting the corresponding values. Group-level analyses were conducted for each region to determine whether the difference in perfusion 1) was significantly different from 0, in the whole population; 2) was significantly different based on intervention (dulaglutide vs placebo) or cigarette smoking status (quitters vs persistent smokers). We used linear regression models as implemented in the lm function in R, with the following covariates: age, baseline carbon monoxide, pack years, and Fagerstroem score. An FDR was applied to correct for multiple comparisons for each analysis.

Structural MRI

T1 images from both sessions were jointly processed using the longitudinal stream of freesurfer v7.2.0.[22] Specifically, an unbiased within-subject template space and image is created using robust, inverse consistent registration.[23] Several processing steps, such as skull stripping, Talairach transforms, atlas registration as well as spherical surface maps and parcellations were then initialized with common information from the within-subject template, significantly increasing reliability and statistical power. The results were individual and reliable volume and cortical thickness estimates.

MRI boilerplate

Anatomical data preprocessing

A total of 2 T1-weighted (T1w) images were found within the input dataset. All of them were corrected for intensity non-uniformity (INU) with N4BiasFieldCorrection distributed with ANTs 2.3.3 (RRID:SCR_004757).[24,25] The T1w-reference was then skull-stripped with a Nipype implementation of the antsBrainExtraction.sh workflow (from ANTs), using OASIS30ANTs as target template. Brain tissue segmentation of CSF, white-matter (WM) and grey-matter (GM) was performed on the brain-extracted T1w using fast (FSL 5.0.9, RRID:SCR_002823).[26] A T1w-reference map was computed after registration of 2 T1w images (after INU-correction) using mri_robust_template (FreeSurfer 6.0.1).[23] Brain surfaces were reconstructed using recon-all (FreeSurfer 6.0.1, RRID:SCR_001847), and the brain mask estimated previously was refined with a custom variation of the method to reconcile ANTs-derived and FreeSurfer-derived segmentations of the cortical grey-matter of Mindboggle (RRID:SCR_002438).[27,28] Volume-based spatial normalization to one standard space (MNI152NLin2009cAsym) was performed through nonlinear registration with antsRegistration (ANTs 2.3.3), using brain-extracted versions of both T1w reference and the T1w template. The following template was selected for spatial normalization: ICBM 152 Nonlinear Asymmetrical template version 2009c (RRID:SCR_008796; TemplateFlow ID: MNI152NLin2009cAsym).[29]

Functional data preprocessing

For each of the task-related and resting-state BOLD runs found per participant (across both sessions), the following preprocessing was performed.

First, a reference volume and its skull-stripped version were generated using a custom methodology of *fMRIPrep*. A B0-nonuniformity map (or *fieldmap*) was estimated based on a phase-difference map calculated with a dual-echo gradient-recall echo sequence, processed with a custom workflow of *SDCFlows* inspired by the epidewarp.fsl script and further improvements in Human Connectome Project Pipelines.[30] The *fieldmap* was then co-registered to the target EPI reference run and converted to a displacements field map (amenable to registration tools such as antsApplyTransforms) with FSL's fugue and other *SDCflows* tools. Based on the estimated susceptibility distortion, a corrected EPI reference was calculated for a more accurate co-registration with the anatomical reference. The BOLD reference was then co-registered to the T1w reference using bbregister (FreeSurfer) which implements boundary-based registration (Greve and Fischl 2009). Co-registration was configured with six degrees of freedom. Head-motion parameters with respect to the BOLD reference (transformation matrices, and six corresponding rotation and translation parameters) are estimated before any spatiotemporal filtering using mcflirt (FSL 5.0.9, [31]). BOLD runs were slice-time corrected using 3dTshift from AFNI (https://afni.nimh.nih.gov/) 20160207 (RRID:SCR_005927): to 1.21s (0.5 of slice acquisition range 0s-2.42s) for the task-based runs, and to 0.864s (0.5 of slice acquisition range 0s-1.73s) for the resting-state runs.[32] The BOLD time-series (including slice-timing correction when applied) were resampled onto their original, native space by applying a single, composite transform to correct for head-motion and susceptibility distortions. These resampled BOLD time-series were referred to as *preprocessed BOLD in original space*, or just *preprocessed BOLD*. The BOLD time-series were resampled into standard space, generating a *preprocessed BOLD run in MNI152NLin2009cAsym space.* Several confounding time-series were calculated based on the *preprocessed BOLD*: FD, the derivative of root mean square variance over voxels (DVARS) and three region-wise global signals. FD was computed using two formulations following Power (absolute sum of relative motions) and Jenkinson (relative root mean square displacement between affines).[31,33] FD and DVARS are calculated for each functional run, both using their implementations in *Nipype* (following the definitions by Power et al.).[33] The three global signals are extracted within the CSF, the WM, and the whole-brain masks. Additionally, a set of physiological regressors were extracted to allow for component-based noise correction (*CompCor*).[34] Principal components are estimated after high-pass filtering the *preprocessed BOLD* time-series (using a discrete cosine filter with 128s cut-off) for the two *CompCor* variants: temporal (tCompCor) and anatomical (aCompCor). tCompCor components are then calculated from the top 2% variable voxels within the brain mask. For aCompCor, three probabilistic masks (CSF, WM, and combined CSF+WM) were generated in anatomical space. The implementation differs from that of Behzadi et al. in that instead of eroding the masks by 2 pixels on BOLD space, the aCompCor masks are subtracted a mask of pixels that likely contain a volume fraction of GM.[34] This mask is obtained by dilating a GM mask extracted from the FreeSurfer's *aseg* segmentation, and it ensures components are not extracted from voxels containing a minimal fraction of GM. Finally, these masks are resampled into BOLD space and binarized by thresholding at 0.99 (as in the original implementation). Components are also calculated separately within the CSF and WM masks. For each CompCor decomposition, the *k* components with the largest singular values were retained, such that the retained components' time series were sufficient to explain 50 percent of variance across the nuisance mask (CSF, WM, combined, or temporal). The remaining components were dropped from consideration. The head-motion estimates calculated in the correction step were also placed within the corresponding confounds file. The confound time series derived from head motion estimates and global signals were expanded with the inclusion of temporal derivatives and quadratic terms for each.[35] Frames that exceeded a threshold of 1.0 mm FD or 1.5 standardized DVARS were annotated as motion outliers. All resamplings were performed with *a single interpolation step* by composing all the pertinent transformations (i.e., head-motion transform matrices, susceptibility distortion correction when available, and co-registrations to

anatomical and output spaces). Gridded (volumetric) resamplings were performed using ANTs, configured with Lanczos interpolation to minimize the smoothing effects of other kernels.[36] Non-gridded (surface) resamplings were performed using mri_vol2surf (FreeSurfer).

Many internal operations of *fMRIPrep* use *Nilearn* 0.6.2 (RRID:SCR_001362), mostly within the functional processing workflow.[37] For more details of the pipeline, see [the section corresponding to workflows in *fMRIPrep*'s documentation].

Tables

Supplementary Table 1: Coordinates of the regions of interest for the cigarette smoking task analysis.

Number	Region	Side	X	Y	Z
1	Anterior cingulate cortex	B	0	24	26
2	Thalamus	B	0	-8	4
3	Amygdala	L	-20	-4	-24
4	Fusiform gyrus	L	-42	-46	-22
5	Inferior frontal gyrus	L	-44	34	12
6	Inferior occipital gyrus	L	-46	-68	-12
7	Inferior parietal lobule	L	-46	-36	44
8	Insula dorsal	L	-40	18	-10
9	Insula posterior	L	-38	-2	0
10	Medial occipital lobe	L	-12	-98	-2
11	Nucleus accumbens	L	-6	8	-8
12	Orbitofrontal gyrus	L	-26	32	-16
13	Posterior cingulate cortex	L	-2	-34	32
14	Precentral gyrus	L	-46	4	28
15	Precuneus	L	-6	-58	38
16	Superior parietal lobule	L	-26	-62	48
17	Thalamus	L	-20	-32	-2
18	Amygdala	R	20	-4	-14
19	Anterior cingulate cortex	R	2	36	10
20	Fusiform gyrus	R	46	-54	-20
21	Inferior occipital gyrus I	R	48	-70	-8
22	Inferior occipital gyrus II	R	32	-92	-8
23	Inferior parietal lobule	R	32	-52	54
24	Insula posterior	R	40	-6	6
25	Insula ventral	R	38	8	-12
26	Insula	R	36	30	-4
27	Precentral gyrus	R	50	6	24
The coordinates (X, Y, Z) are in MNI space. Abbreviations: B = bilateral, L = left, MNI = Montreal Neurological Institute, R = right.					

Supplementary Table 2: Baseline characteristics for women and men separately.

	Women	Men
**Number of participants**	**N = 41**	**N = 29**
Age (years), mean (SD)	36.54 (10.72)	39.55 (11.04)
Weight (kg), mean (SD)	70.08 (10.66)	93.47 (15.39)
BMI (kg/m^2), mean (SD)	24.46 (3.23)	28.63 (4.03)
HbA1c (%), median [IQR]	5.20 [5.00, 5.50]	5.40 [5.30, 5.70]
Caucasian, n (%)	39 (95.1)	29 (100.0)
Handedness, n (%)<br>- right<br>- left	 - 39 (95)<br>- 2 (5)	 - 28 (97)<br>- 1 (3)
Pack years, median [IQR]	14.00 [9.00, 22.00]	13.00 [9.00, 26.00]
Cigarettes per day, median [IQR]	20.00 [15.00, 20.00]	20.00 [15.00, 20.00]
End-expiratory exhaled carbon monoxide (ppm), median [IQR]	15.50 [12.75, 23.00]	19.00 [13.00, 25.00]
Fagerstroem total sum score, mean (SD)	6.82 (1.11)	6.83 (1.26)
Craving for cigarette smoking QSU-G (total sum score), mean (SD)	117.40 (39.34)	114.14 (38.55)
Abbreviations: BMI = Body mass index, HbA1c = hemoglobin A1c, IQR = interquartile range, QSU-G = Questionnaire on Smoking urges - German version, SD = Standard deviation, n = number, VAS = Visual Analog Scale.		

Supplementary Table 3: QSU-G total sum scores based on intervention at baseline and the 12-week visit.

	**Baseline** N = 70, median (IQR)	**12-week visit** N = 69, median (IQR)	**Estimated mean difference** (95% CI)	**p-value**
**Dulaglutide**	108.50 (86.25, 137.75)	49.00 (32.50, 66.00)	-9.10 (-22.26, 3.95)	0.18
**Placebo**	123.87 (83.50, 156.50)	51.50 (38.00, 68.50)		
Abbreviations: IQR = interquartile range.				

Supplementary Table 4: Craving for cigarette smoking (VAS) during functional MRI based on intervention at baseline and the 12-week visit.

**Dulaglutide**, median (IQR)		**Placebo**, median (IQR)		**Rate ratio for intervention** (95% CI), p-value	**Effect size intervention** (95% CI)	**Rate ratio for smoking cues** (95% CI), p-value	**Effect size stimulus cues** (95% CI)
				**Baseline**			
Neutral videos, N = 34	Smoking videos, N = 34	Neutral videos, N = 36	Smoking videos, N = 36				
2 (1, 2.5)	5.5 (5, 6)	2 (1, 3)	5.5 (5, 6)	0.98 (0.86, 1.13); 0.81	-0.02 (-0.15, 0.12)	1.97 (1.86, 2.09); <0.001	0.68 (0.62, 0.73)
				**12-week visit**			
Neutral videos, N = 26	Smoking videos, N = 26	Neutral videos, N = 31	Smoking videos, N = 31				
1 (1, 1)	1 (1, 2)	1 (1, 1)	1.5 (1, 3)	0.85 (0.675, 1.07); 0.15	-0.16 (-0.39, 0.06)	1.573 (1.44, 1.72); <0.001	0.45 (0.36, 0.54)

Displayed are results from a generalized mixed effect model with Poisson distribution. Model effect size was estimated using package "effectsize" in R.
In a generalized linear mixed effect model with Poisson distribution, there was no difference based on intervention (rate ratio 0.9365 (95% CI 0.814, 1.08); p=0.35), response was significantly lower for both the 12-week visit (rate ratio 0.48 (95% CI 0.45, 0.50); p<0.001) and cigarette smoking videos (rate ratio 1.8459 (95% CI 1.76, 1.94); p<0.001). Effect size was -0.07 (-0.20, 0.07) for intervention, -0.37 (-0.39, -0.34) for session and 0.61 (0.56, 0.66) for smoking stimulus.
Abbreviations: IQR = interquartile range, CI = Confidence interval.

Supplementary Table 5: Craving for cigarette smoking (VAS) during functional MRI based on cigarette smoking status at baseline and the 12-week visit.

**Quitters** median (IQR)		**Persistent smokers** median (IQR)		**Rate ratio for smoking status** (95% CI), p-value	**Effect size smoking status** (95% CI)	**Rate ratio for smoking cues** (95% CI), p-value	**Effect size smoking cues** (95% CI)
				**Baseline**			
Neutral videos, N = 39	Smoking videos, N = 39	Neutral videos, N = 31	Smoking videos, N = 31				
2 (1, 1.5)	5.5 (5, 6)	2, (1.25, 3)	6 (5, 6.5)	1.06 (0.93, 1.22); 0.39	0.06 (-0.08, 0.20)	1.97 (1.86, 2.09); <0.001	0.87 (0.69, 1.06)
				**12-week visit**			
Neutral videos, N = 36	Smoking videos, N = 36	Neutral videos, N = 21	Smoking videos, N = 21				
1 (1, 1)	1 (1, 2)	1 (1, 1)	2 (1, 4)	1.33 (1.06, 1.67); 0.013	0.28 (0.06, 0.51)	1.57 (1.44, 1.72); <0.001	0.45 (0.36, 0.54)

Displayed are results from a generalized linear model with Poisson distribution. Model effect size was estimated using package "effectsize" in R. In a generalized linear mixed effect model with Poisson distribution, there was no difference based on smoking status (rate ratio 1.11 (95% CI 0.97, 1.28); p=0.12), but when exposed to smoking cues craving ratings were higher (rate ratio 1.85 (95% CI 1.76, 1.94); p<0.001) but overall lower at the 12-week visit (rate ratio 0.48 (95% CI 0.45, 0.50); p<0.001). Effect size was 0.11 (-0.03, 0.24) for smoking status, 0.61 (0.56, 0.66) for smoking stimulus and -0.37 (-0.39, -0.34) for session.
Abbreviations: IQR = interquartile range, CI = Confidence interval.

Supplementary Table 6: N-Back task based on intervention at the 12-week visit.

N-back task	**Dulaglutide**, N = 26, median (IQR)	**Placebo**, N = 31, median (IQR)	**Estimated mean difference intervention** (95% CI)	**p-value**	**Effect size** (95% CI)
**Reaction time**					
0-Back	436 (387, 510)	444 (414, 550)	6.38 (-40.66, 53.43)	0.79	0.07 (-0.45, 0.60)
1/2-Back	448 (399, 621)	465 (421 689)	29.83 (-19.73, 79.38)	0.23	0.34 (-0.22, 0.88)
**Accuracy**					
0-Back	0.957 (0.943, 0.986)	0.943 (0.943, 0.971)	-0.003 (-0.019, 0.013)	0.69	-0.11 (-0.63, 0.41)
1/2-Back	0.907 (0.9, 0.929)	0.9 (0.886, 0.936)	-0.004 (-0.017, 0.008)	0.51	-0.18 (-0.70, 0.35)

In a mixed effect model, no significant differences were observed between dulaglutide and placebo (reaction time: dulaglutide estimated difference -19.0 (95% CI -51.33, 13.52); p=0.25, effect size -0.22 (-0.58, 0.15)), accuracy: dulaglutide estimated difference 0.003 (95% CI -0.005, 0.012), p=0.46, effect size 0.09 (-0.15, 0.33)). Abbreviations: IQR = interquartile range, CI = Confidence interval.

Supplementary Table 7: N-Back task based on cigarette smoking status at the 12-week visit.

N-Back task	**Quitters,** N = 36, median (IQR),	**Persistent smoker,** N = 21, median (IQR)	**Estimated mean difference smoking status** (95% CI)	**p-value**	**Effect size** (95% CI)
**Reaction time**					
0-Back	418 (392, 499)	464 (426, 550)	-42.46 (-92.55, 7.63)	0.09	-0.51 (-1.06, 0.04)
1/2-Back	457 (404, 647)	484 (421, 644)	-19.17 (-75.34, 37.10)	0.49	-0.21 (-0.79, 0.37)
**Accuracy**					
0-Back	0.95 (0.943 0.986)	0.943 (0.943 0.971)	0.002 (-0.015, 0.020)	0.78	0.08 (-0.45, 0.62)
1/2-Back	0.914 (0.9, 0.932)	0.9 (0.871, 0.929)	0.008 (-0.005, 0.021)	0.21	0.34 (-0.20, 0.88)

In a linear mixed effect model, we found slower reaction time in persistent smokers (reaction time: persistent smokers estimated difference 40.49 (95% CI 8.67, 72.36), p=0.02, effect size 0.46 (0.10, 0.82); accuracy: persistent smokers estimated difference -0.006 (95% CI -0.014, 0.003), p=0.18, effect size -0.16 (-0.40, 0.08)).
Abbreviations: IQR = interquartile range, CI = Confidence interval.

Supplementary Table 8: Regions of interest analysis for the cigarette smoking task.

A

**Region**	**Baseline**			**12-week visit VS baseline**		
	T	p	pFDR	T	p	pFDR
B anterior cingulate cortex	0.54	0.59	0.92	-2.54	0.01	0.26
B thalamus	0.28	0.78	0.93	-1.26	0.22	0.30
L amygdala	-0.01	0.99	0.99	-2.43	0.02	0.26
L fusiform gyrus	-1.05	0.30	0.61	-0.55	0.58	0.74
L inferior frontal gyrus	-1.31	0.20	0.50	0.37	0.71	0.83
L inferior occipital gyrus	-1.03	0.31	0.61	-1.91	0.06	0.28
L inferior parietal lobule	-4.60	2.23E-05	3.12E-04	0.10	0.92	0.95
L insula dorsal	0.26	0.79	0.93	-1.55	0.13	0.28
L insula posterior	-2.03	0.05	0.15	-1.42	0.16	0.28
L medial occipital lobe	-3.28	1.76E-03	0.02	0.06	0.96	0.96
L nucleus accumbens	-0.07	0.94	0.99	-1.58	0.12	0.28
L orbitofrontal gyrus	0.30	0.76	0.93	0.26	0.80	0.87
L posterior cingulate cortex	0.49	0.62	0.92	-1.32	0.19	0.30
L precentral gyrus	-2.28	0.03	0.09	-0.25	0.81	0.87
L precuneus	2.60	0.01	0.05	-0.89	0.38	0.51
L superior parietal lobule	0.06	0.95	0.99	-0.51	0.61	0.74
L thalamus	-0.35	0.73	0.93	-2.20	0.03	0.28
R amygdala	-0.99	0.33	0.61	-1.65	0.11	0.28
R anterior cingulate cortex	0.39	0.70	0.93	-1.97	0.05	0.28
R fusiform gyrus	-0.04	0.97	0.99	-1.75	0.09	0.28
R inferior occipital gyrus I	-5.41	1.15E-06	3.21E-05	-1.64	0.11	0.28
R inferior occipital gyrus II	0.72	0.48	0.83	-1.41	0.17	0.28
R inferior parietal lobule	-3.12	2.76E-03	0.02	-1.39	0.17	0.28

R insula	-1.01	0.32	0.61	-1.30	0.20	0.30
R insula posterior	-3.17	2.42E-03	0.02	-1.59	0.12	0.28
R insula ventral	-0.61	0.54	0.90	-2.09	0.04	0.28
R precentral gyrus	-2.46	0.02	0.07	-1.47	0.15	0.28
B medial orbitofrontal cortex	1.58	0.12	0.34	-1.42	0.16	0.28

B

**Region**	**delta: smoking status**			**delta: intervention**		
	T	p	pFDR	T	p	pFDR
B anterior cingulate cortex	-1.40	0.17	0.83	0.06	0.95	0.98
B thalamus	-0.82	0.42	0.83	-0.18	0.86	0.97
L amygdala	0.86	0.40	0.83	2.59	0.01	0.19
L fusiform gyrus	1.08	0.29	0.83	-0.02	0.98	0.98
L inferior frontal gyrus	-0.95	0.35	0.83	-0.92	0.36	0.83
L inferior occipital gyrus	-0.34	0.73	0.90	1.12	0.27	0.69
L inferior parietal lobule	-0.25	0.80	0.90	0.19	0.85	0.97
L insula dorsal	0.03	0.98	1.00	0.23	0.82	0.97
L insula posterior	-1.18	0.24	0.83	-0.12	0.91	0.98
L medial occipital lobe	0.38	0.71	0.90	0.46	0.65	0.97
L nucleus accumbens	1.25	0.22	0.83	1.61	0.11	0.45
L orbitofrontal gyrus	0.48	0.63	0.90	0.35	0.73	0.97
L posterior cingulate cortex	0.89	0.38	0.83	0.34	0.74	0.97
L precentral gyrus	-1.02	0.31	0.83	-0.23	0.82	0.97
L precuneus	0.73	0.47	0.83	2.11	0.04	0.22
L superior parietal lobule	0.31	0.76	0.90	0.66	0.51	0.97
L thalamus	0.73	0.47	0.83	0.36	0.72	0.97
R amygdala	-0.72	0.48	0.83	2.40	0.02	0.19

R anterior cingulate cortex	-1.21	0.23	0.83	2.44	0.02	0.19
R fusiform gyrus	0.61	0.55	0.90	0.88	0.38	0.83
R inferior occipital gyrus I	-0.83	0.41	0.83	2.19	0.03	0.22
R inferior occipital gyrus II	-0.01	1.00	1.00	0.43	0.67	0.97
R inferior parietal lobule	-1.35	0.18	0.83	1.35	0.18	0.57
R insula	0.30	0.77	0.90	-1.55	0.13	0.45
R insula posterior	-0.48	0.63	0.90	1.22	0.23	0.64
R insula ventral	-0.12	0.91	0.98	0.42	0.68	0.97
R precentral gyrus	-0.31	0.76	0.90	0.49	0.63	0.97
B medial orbitofrontal cortex	2.22	0.03	0.83	1.86	0.07	0.32

T-values, p-values, and FDR-corrected p-values are reported for the following linear models: (A) baseline mean activation (“Baseline”), difference between 12-week visit and baseline (“12-week visit VS baseline”); (B) difference from baseline to the 12-week visit based on smoking status (“Delta: smoking status”). In this context, positive T-values represent a greater difference in activation for persistent smoker’s vs quitters. The last model investigates the difference from baseline to the 12-week visit based on intervention group (“Delta: intervention”). In this context, positive T-values represent a greater difference in activation for the placebo vs dulaglutide group. Abbreviations: B = bilateral, L = left, R = right.

Supplementary Table 9: Gray matter brain volume based on intervention at baseline and the 12-week visit.

Gray matter brain volume (dm^3)	**Dulaglutide**, N = 28, mean ±SD	**Placebo**, N = 30, mean ±SD,	**p-value (raw)**	**p-value (adjusted)**
**Baseline**				
Left frontal lobe	97489.3 (9527.5)	93276.7 (9452.6)	0.097	0.260
Left parietal lobe	65532.8 (7130.4)	64666.9 (5980.2)	0.620	0.646
Left temporal lobe	59364.1 (6086.5)	58196.4 (6040.0)	0.467	0.584
Left occipital lobe	24843.4 (3235.5)	23970.7 (2633.2)	0.267	0.414
Left insula	7604.4 (874.9)	7232.5 (834.0)	0.104	0.260
Left thalamus	8557.5 (891.8)	8450.9 (831.0)	0.640	0.798
Left caudate	3843.8 (395.6)	3855.6 (458.4)	0.916	0.946
Left putamen	5592.3 (625.9)	5352.8 (530.3)	0.123	0.672
Left pallidum	2231.2 (221.5)	2174.1 (272.2)	0.384	0.672
Left hippocampus	4549.6 (427.9)	4461.0 (535.6)	0.488	0.732
Left amygdala	1721.4 (215.4)	1643.7 (208.3)	0.169	0.672
Right frontal lobe	97083.4 (9141.9)	92943.2 (8976.6)	0.088	0.260
Right parietal lobe	67814.2 (7097.7)	65010.5 (7035.6)	0.137	0.274
Right temporal lobe	57599.8 (5974.4)	56905.0 (5430.0)	0.646	0.646
Right occipital lobe	26218.5 (3611.8)	25326.9 (2618.7)	0.290	0.414
Right insula	7390.6 (786.3)	6989.4 (802.4)	0.060	0.260
Right thalamus	8462.6 (770.2)	8449.7 (673.3)	0.946	0.946
Right caudate	4040.4 (418.9)	3988.3 (491.3)	0.665	0.798
Right putamen	5589.5 (664.3)	5378.5 (473.8)	0.173	0.672
Right pallidum	2131.5 (275.9)	2065.7 (304.6)	0.392	0.672
Right hippocampus	4661.0 (489.7)	4547.0 (496.9)	0.383	0.672
Right amygdala	1976.0 (209.8)	1908.0 (240.8)	0.256	0.672
**12-week visit**				
Left frontal lobe	97404.4 (9408.8)	92360.2 (9810.7)	0.051	0.174
Left parietal lobe	65393.3 (7148.4)	64176.2 (6046.5)	0.488	0.542
Left temporal lobe	59043.7 (5792.7)	57683.2 (6048.8)	0.385	0.481
Left occipital lobe	24594.9 (3125.2)	23798.4 (2801.7)	0.313	0.447
Left insula	7601.8 (876.1)	7191.7 (807.2)	0.070	0.174
Left thalamus	8558.5 (858.2)	8415.3 (864.8)	0.529	0.712
Left caudate	3810.7 (388.8)	3811.3 (465.3)	0.996	0.996
Left putamen	5582.1 (601.0)	5344.9 (547.1)	0.123	0.642
Left pallidum	2218.7 (230.5)	2171.4 (256.5)	0.463	0.712
Left hippocampus	4541.9 (418.1)	4468.3 (534.6)	0.560	0.712
Left amygdala	1718.9 (220.1)	1642.6 (205.8)	0.179	0.642
Right frontal lobe	96848.0 (8941.8)	92111.5 (9321.6)	0.053	0.174
Right parietal lobe	67383.2 (6882.8)	64568.1 (7222.8)	0.134	0.268
Right temporal lobe	57137.1 (5672.8)	56518.5 (5222.8)	0.668	0.668
Right occipital lobe	26007.1 (3503.6)	25148.0 (2755.9)	0.306	0.447
Right insula	7322.7 (793.5)	6893.6 (793.5)	0.044	0.174
Right temporal lobe	57137.1 (5672.8)	56518.5 (5222.8)	0.667	0.668
Right thalamus	8453.3 (735.6)	8421.9 (720.1)	0.870	0.949

Right caudate	4013.9 (420.8)	3949.1 (496.2)	0.593	0.712
Right putamen	5575.7 (659.7)	5370.1 (503.2)	0.190	0.642
Right pallidum	2122.6 (266.9)	2060.5 (286.1)	0.396	0.712
Right hippocampus	4661.9 (469.6)	4529.6 (490.5)	0.298	0.712
Right amygdala	1969.7 (203.1)	1896.9 (238.1)	0.214	0.642
Estimated total intracranial	1650419.7 (169470.0)	1579176.6 (186440.2)	0.134	0.400
P-values adjusted for multiple testing using false discovery rate.				

Supplementary Table 10: Gray matter brain volume according to cigarette smoking status at baseline and the 12-week visit.

**Gray matter brain volume (dm^3)**	**Quitters,** N = 35, mean ±SD	**Smokers,** N = 23, mean ±SD	**p-value (raw)**	**p-value (adjusted)**
**Baseline**				
Left frontal lobe	95928.9 (9969.0)	94369.3 (9259.8)	0.546	0.988
Left parietal lobe	65095.5 (6439.1)	65068.7 (6779.1)	0.988	0.988
Left temporal lobe	59136.0 (6280.4)	58188.2 (5738.2)	0.556	0.988
Left occipital lobe	24312.3 (2975.7)	24513.3 (2962.8)	0.802	0.988
Left insula	7405.4 (950.4)	7422.1 (742.5)	0.941	0.988
Left thalamus	8433.4 (829.9)	8607.4 (900.2)	0.462	0.924
Left caudate	3771.7 (455.1)	3968.9 (352.9)	0.070	0.834
Left putamen	5459.3 (611.7)	5482.2 (557.3)	0.884	0.964
Left pallidum	2200.5 (230.1)	2203.3 (279.6)	0.969	0.969
Left hippocampus	4521.0 (506.2)	4477.5 (459.3)	0.736	0.954
Left amygdala	1675.4 (221.7)	1690.2 (205.0)	0.795	0.954
Right frontal lobe	95704.1 (9638.6)	93782.1 (8611.9)	0.432	0.988
Right parietal lobe	65979.6 (7458.5)	66949.0 (6759.1)	0.610	0.988
Right temporal lobe	57707.1 (5936.5)	56530.3 (5258.4)	0.432	0.988
Right occipital lobe	25851.3 (2942.6)	25614.3 (3486.5)	0.789	0.988
Right insula	7190.9 (917.6)	7171.2 (641.8)	0.924	0.988
Right thalamus	8389.5 (646.4)	8557.1 (813.8)	0.411	0.924
Right caudate	3969.9 (495.1)	4079.7 (385.9)	0.348	0.924
Right putamen	5496.0 (617.2)	5456.5 (526.3)	0.795	0.954
Right pallidum	2073.4 (270.4)	2134.2 (321.4)	0.458	0.924
Right hippocampus	4563.9 (528.1)	4659.9 (437.6)	0.455	0.924
Right amygdala	1952.0 (222.6)	1923.8 (237.5)	0.652	0.954
**12-Week visit**				
Left frontal lobe	95183.7 (10384.2)	94204.3 (9220.4)	0.708	0.986
Left parietal lobe	64653.1 (6542.2)	64932.1 (6759.0)	0.877	0.986
Left temporal lobe	58699.9 (6206.9)	57792.2 (5528.9)	0.563	0.986
Left occipital lobe	24085.2 (3064.3)	24331.6 (2863.8)	0.757	0.986
Left insula	7391.1 (947.8)	7387.3 (723.4)	0.986	0.986
Left thalamus	8407.4 (811.2)	8601.7 (928.5)	0.417	0.845
Left caudate	3718.2 (438.7)	3952.2 (372.7)	0.034	0.407
Left putamen	5444.0 (591.5)	5482.9 (577.3)	0.805	0.957
Left pallidum	2198.4 (231.9)	2187.9 (265.0)	0.877	0.957

Left hippocampus	4528.2 (500.9)	4466.8 (452.5)	0.630	0.888
Left amygdala	1669.6 (221.7)	1694.4 (206.8)	0.666	0.888
Right frontal lobe	94966.5 (10056.1)	93533.1 (8357.9)	0.559	0.986
Right parietal lobe	65440.8 (7501.0)	66667.2 (6649.8)	0.517	0.986
Right temporal lobe	57253.4 (5715.1)	56153.3 (4945.9)	0.440	0.986
Right occipital lobe	25628.2 (2943.0)	25463.2 (3487.0)	0.852	0.986
Right insula	7092.0 (920.2)	7114.0 (644.3)	0.915	0.986
Right thalamus	8361.2 (651.5)	8552.4 (818.0)	0.352	0.845
Right caudate	3925.3 (490.1)	4064.1 (401.7)	0.244	0.845
Right putamen	5467.2 (622.6)	5472.5 (544.6)	0.973	0.973
Right pallidum	2066.9 (264.8)	2126.4 (295.5)	0.439	0.845
Right hippocampus	4558.4 (503.8)	4646.7 (449.5)	0.489	0.845
Right amygdala	1948.4 (226.6)	1907.1 (220.0)	0.493	0.845
Estimated total intracranial	1616820.6 (179311.7)	1608623.1 (186214.0)	0.867	0.400
P-values were adjusted for multiple testing using false discovery rate.				

Supplementary Table 11: Regression model for cortical and subcortical grey matter brain volume based on intervention at baseline and the 12-week visit.

Gray matter brain volume (dm^3)	**Dulaglutide**, N = 28, mean ±SD	**Placebo**, N = 30, mean ±SD,	**Estimated difference (95% CI)**	**p-value (raw)**	**p-value (adjusted)**
Left frontal lobe	-85.0 (1844.2)	-916.5 (1599.4)	1841 (-143.9, 1714.9)	0.349	0.935
Left parietal lobe	-139.5 (1325.1)	-490.7 (1170.1)	-857.7 (-3458.8, 1743.5)	0.529	0.935
Left temporal lobe	-320.4 (897.6)	-513.3 (823.7)	-511.4 (-2768.9, 1746.1)	0.665	0.935
Left occipital lobe	-248.5 (489.2)	-172.3 (518.0)	262.0 (-1123.8, 1647.8)	0.718	0.935
Left insula	-2.7 (166.9)	-40.8 (152.4)	197.5 (-190.8, 585.8)	0.332	0.935
Left thalamus	1.0 (121.8)	-35.6 (129.6)	-44.74 (-437.4, 347.9)	0.827	0.935
Left caudate	-33.1 (61.5)	-44.3 (89.5)	-79.24 (-283.5, 125.0)	0.459	0.935
Left putamen	-10.2 (109.8)	-7.9 (101.0)	100.8 (-156.3, 357.8)	0.454	0.935
Left pallidum	-12.5 (40.9)	-2.7 (67.5)	4.3 (-107.3, 115.8)	0.941	0.941
Left hippocampus	-7.7 (79.7)	7.3 (71.8)	-21.7 (-241.4, 198.1)	0.850	0.935
Left amygdala	-2.5 (67.5)	-1.1 (54.5)	26.6 (-63.4, 116.6)	0.5722	0.935
Right frontal lobe	-235.4 (1922.2)	-831.7 (1720.4)	1754 (-1714.8, 5223.1)	0.335	0.914
Right parietal lobe	-430.9 (1352.6)	-442.4 (1400.0)	722.3 (-1961.0 3405.6)	0.607	0.914
Right temporal lobe	-462.8 (860.4)	-386.5 (686.8)	-964.8 (-3023.8, 1094.2)	0.372	0.914
Right occipital lobe	-211.4 (593.6)	-178.8 (557.2)	237.4 (-1257.0, 1731.8)	0.761	0.914
Right insula	-68.0 (157.9)	-95.9 (163.4)	235.1 (-123.7, 593.9)	0.213	0.914
Right thalamus	-9.3 (102.3)	-27.8 (106.4)	-112.1 (-452.3, 228.0)	0.529	0.914
Right caudate	-26.4 (63.6)	-39.2 (70.6)	-26.7 (-242.6, 189.1)	0.813	0.914
Right putamen	-13.8 (100.4)	-8.4 (86.7)	69.3 (-187.5, 326.1)	0.606	0.914
Right pallidum	-8.9 (44.4)	-5.2 (61.0)	14.8 (-118.6, 148.1)	0.832	0.914
Right hippocampus	0.9 (63.6)	-17.4 (75.5)	12.88 (-213.9, 239.7)	0.914	0.914
Right amygdala	-6.2 (56.3)	-11.2 (48.1)	11.6 (-86.7, 109.8)	0.822	0.914
Regression models were adjusted for sex and estimated total intracranial volume. P-values were adjusted for multiple testing using false discovery rate. Abbreviations: CI = Confidence interval.					

Supplementary Table 12: Regression model for cortical and subcortical grey matter brain volume based on cigarette smoking status at baseline and the 12-week visit.

**Gray matter brain volume (dm^3)**	**Quitters,** N = 35, mean ±SD	**Smokers,** N = 23, mean ±SD	**Estimated difference** (95% CI)	**p-value (raw)**	**p-value (adjusted)**
Left frontal lobe	-745.1 (1949.6)	-165.0 (1384.0)	-1028 (-4753.8, 2698.3)	0.598	0.935
Left parietal lobe	-442.4 (1292.0)	-136.7 (1184.0)	291.6 (-2291.6, 2874.7)	0.829	0.935
Left temporal lobe	-436.0 (888.8)	-396.0 (828.2)	-755.1 (-2984.8, 1474.6)	0.518	0.935
Left occipital lobe	-227.1 (513.7)	-181.8 (492.0)	280.1 (-1091.3, 1651.5)	0.696	0.935
Left insula	-14.3 (184.3)	-34.8 (113.9)	19.9 (-367.8, 407.6)	0.922	0.935
Left thalamus	-26.0 (111.7)	-5.7 (147.2)	205.5 (-179.6, 590.6)	0.309	0.935
Left caudate	-53.5 (59.2)	-16.6 (94.9)	224.9 (30.4, 419.4)	**0.031**	0.935
Left putamen	-15.3 (88.7)	0.7 (126.2)	41.8 (-213.7, 297.4)	0.754	0.935
Left pallidum	-2.1 (36.9)	-15.5 (76.9)	2.4 (-108.0, 112.8)	0.967	0.941
Left hippocampus	7.2 (79.3)	-10.8 (69.5)	-41.7 (-2589.0, 175.6)	0.714	0.935
Left amygdala	-5.7 (49.5)	4.3 (75.2)	25.4 (-63.7, 114.5)	0.586	0.935
Right frontal lobe	-737.6 (1983.7)	-249.0 (1561.4)	-1429 (-4872.7, 2013.9)	0.428	0.935
Right parietal lobe	-538.8 (1484.0)	-281.7 (1176.5)	1296 (-1344.6, 3937.1)	0.350	0.935
Right temporal lobe	-453.8 (751.2)	-377.0 (811.4)	-96.2 (-3000.3, 1075.4)	0.368	0.935
Right occipital lobe	-223.1 (523.0)	-151.2 (645.4)	-162.5 (-1642.4, 1317.3)	0.834	0.935
Right insula	-98.9 (176.4)	-57.2 (130.9)	16.4 (-343.9, 376.7)	0.931	0.935
Right thalamus	-28.2 (81.0)	-4.7 (132.3)	195.0 (-139.0, 529.0)	0.267	0.935
Right caudate	-44.5 (69.3)	-15.6 (60.9)	134.2 (-76.7, 345.0)	0.227	0.935
Right putamen	-28.8 (85.0)	16.0 (99.4)	-5.5 (-260.3, 249.3)	0.967	0.935
Right pallidum	-6.4 (53.9)	-7.8 (53.3)	66.7 (-64.1, 197.6)	0.331	0.941
Right hippocampus	-5.5 (72.6)	-13.2 (67.2)	98.3 (-124.8, 321.3)	0.401	0.935
Right amygdala	-3.6 (40.9)	-16.6 (65.3)	-31.6 (-128.5, 65.4)	0.534	0.935

Regression models were adjusted for sex and estimated total intracranial volume. P-values were adjusted for multiple testing using false discovery rate. Abbreviations: CI = Confidence interval.

Figures

Supplementary Figure 1: Trial procedures and timeline.

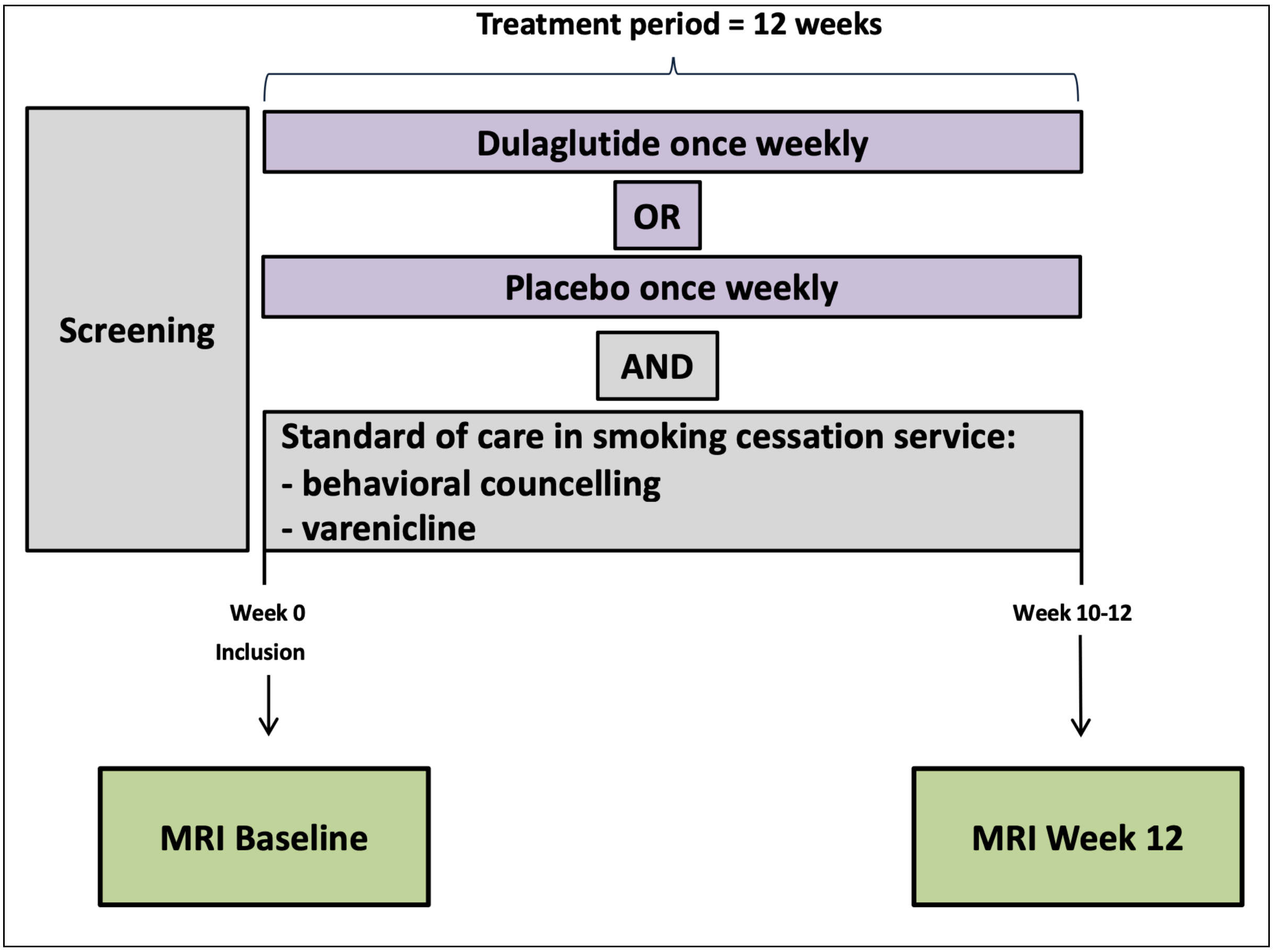

Supplementary Figure 2: Regions of interest for the cigarette smoking task.

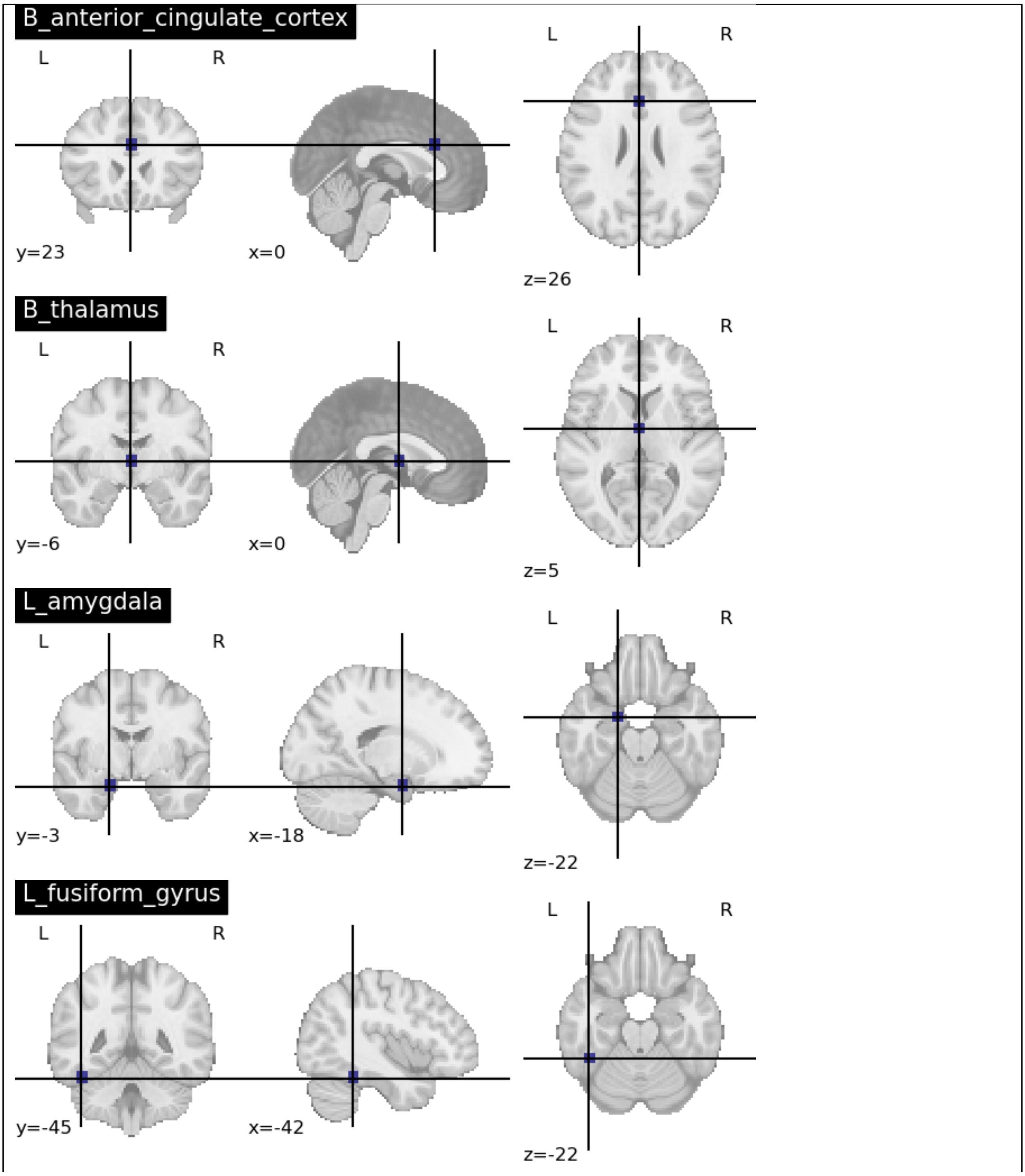

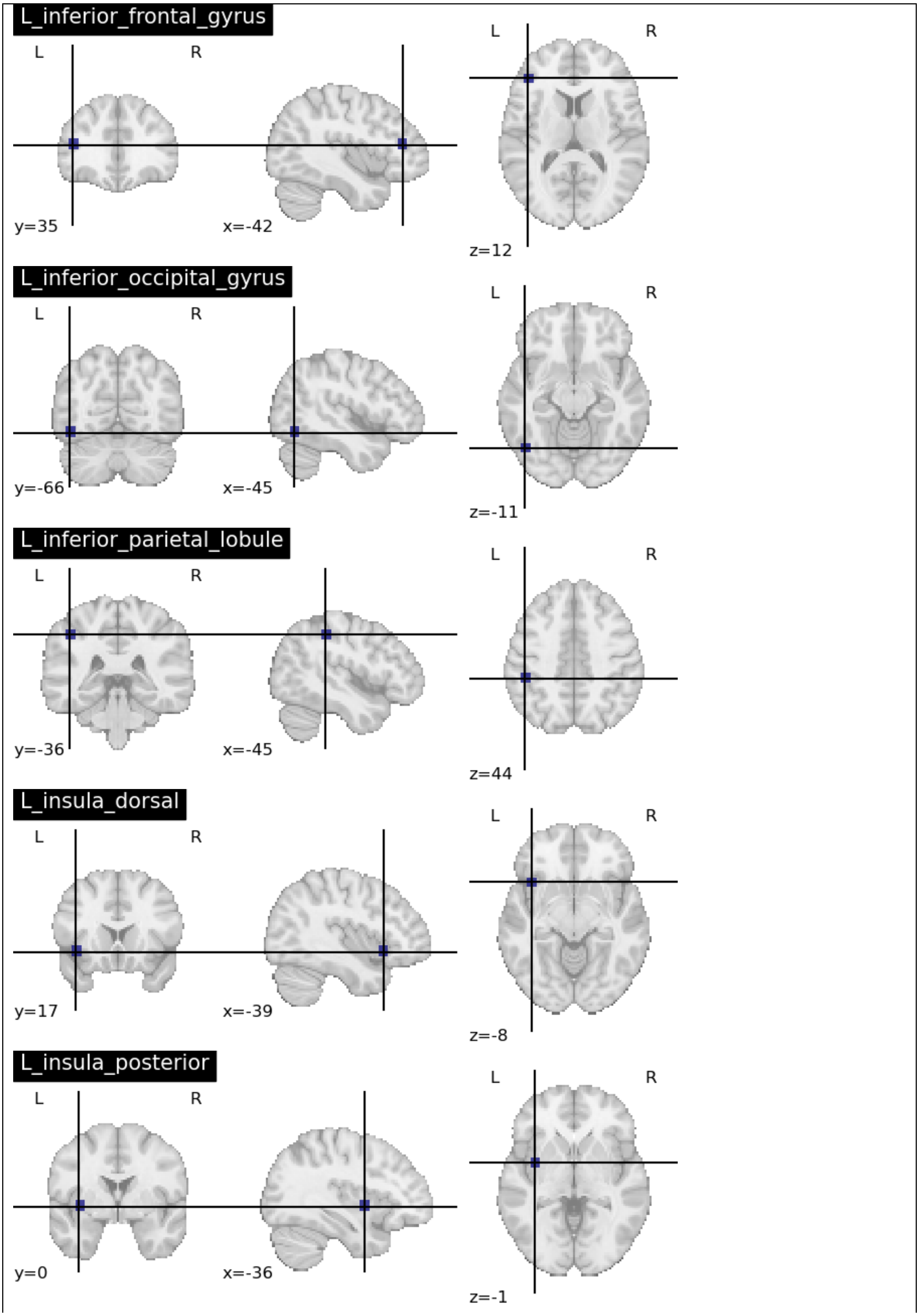
L_inferior_frontal_gyrus
L
R
y=35
x=-42
L
R
z=12
L_inferior_occipital_gyrus
L
R
y=-66
x=-45
L
R
z=-11
L_inferior_parietal_lobule
L
R
y=-36
x=-45
L
R
z=44
L_insula_dorsal
L
R
y=17
x=-39
L
R
z=-8
L_insula_posterior
L
R
y=0
x=-36
L
R
z=-1

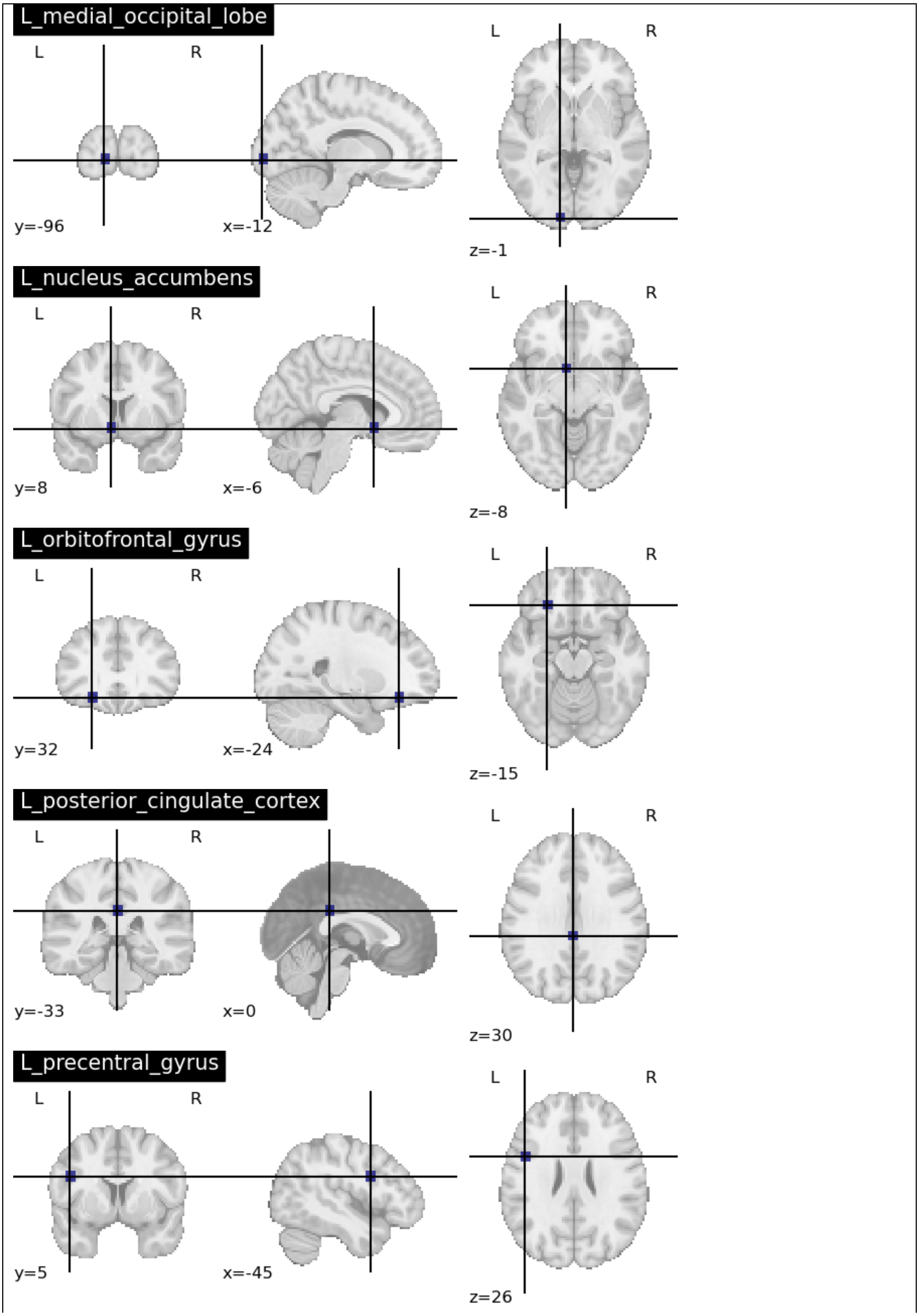
L_medial_occipital_lobe
L
R
y=-96
x=-12
L
R
z=-1
L_nucleus_accumbens
L
R
y=8
x=-6
L
R
z=-8
L_orbitofrontal_gyrus
L
R
y=32
x=-24
L
R
z=-15
L_posterior_cingulate_cortex
L
R
y=-33
x=0
L
R
z=30
L_precentral_gyrus
L
R
y=5
x=-45
L
R
z=26

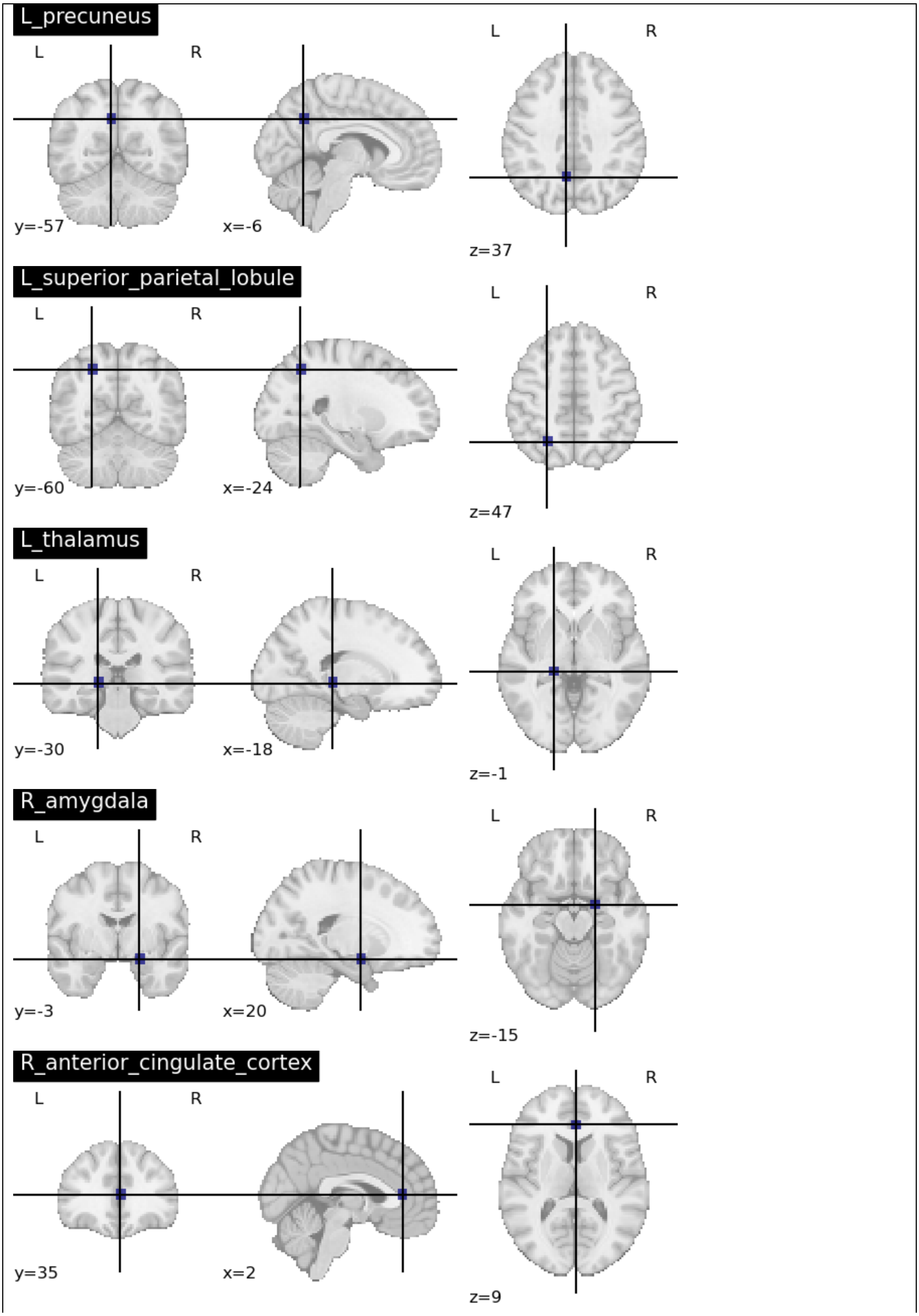
L_precuneus
L R
L R
y=-57
x=-6
z=37
L_superior_parietal_lobule
L R
L R
y=-60
x=-24
z=47
L_thalamus
L R
L R
y=-30
x=-18
z=-1
R_amygdala
L R
L R
y=-3
x=20
z=-15
R_anterior_cingulate_cortex
L R
L R
y=35
x=2
z=9

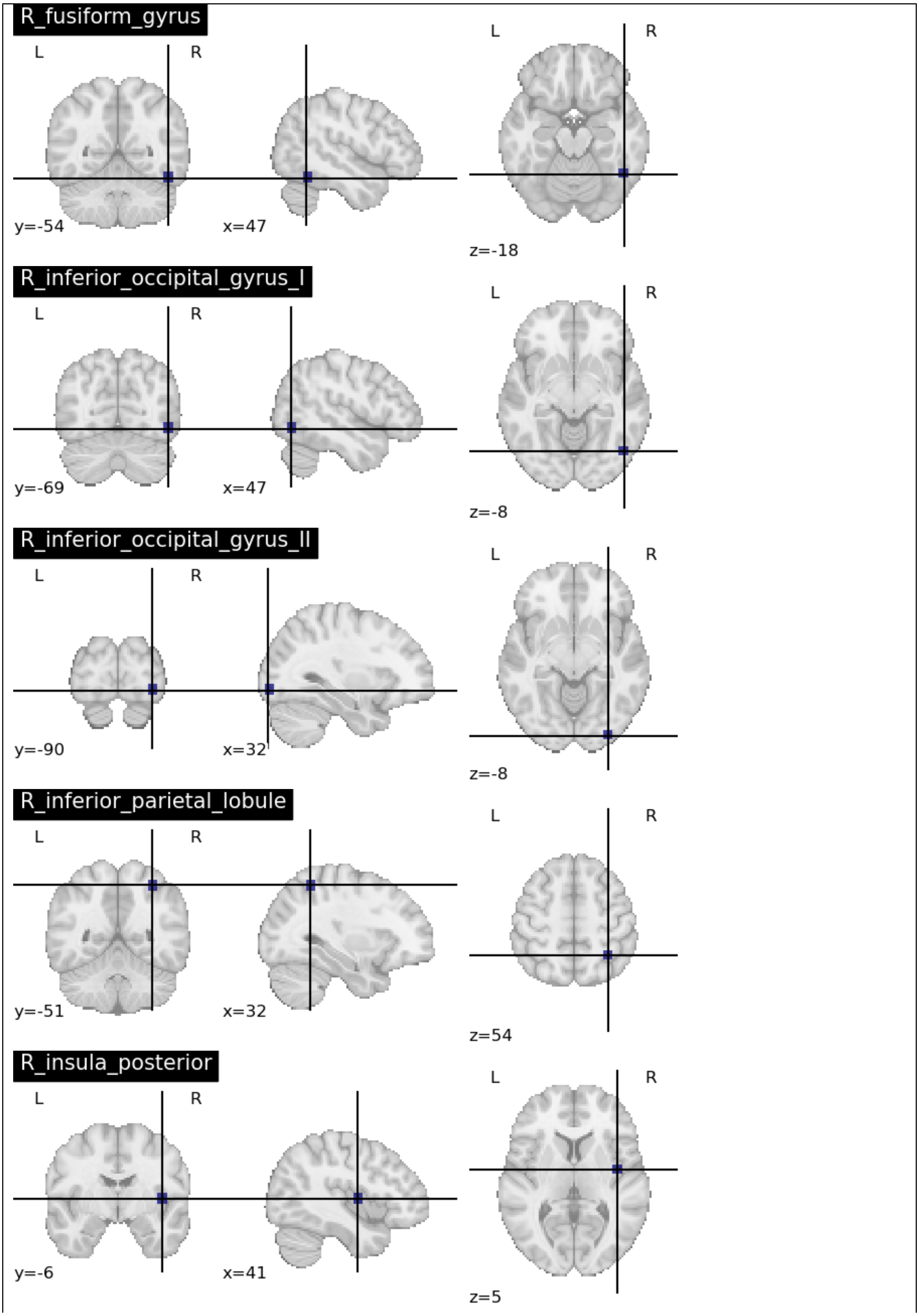
R_fusiform_gyrus
L
R
y=-54
x=47
L
R
z=-18
R_inferior_occipital_gyrus_I
L
R
y=-69
x=47
L
R
z=-8
R_inferior_occipital_gyrus_II
L
R
y=-90
x=32
L
R
z=-8
R_inferior_parietal_lobule
L
R
y=-51
x=32
L
R
z=54
R_insula_posterior
L
R
y=-6
x=41
L
R
z=5

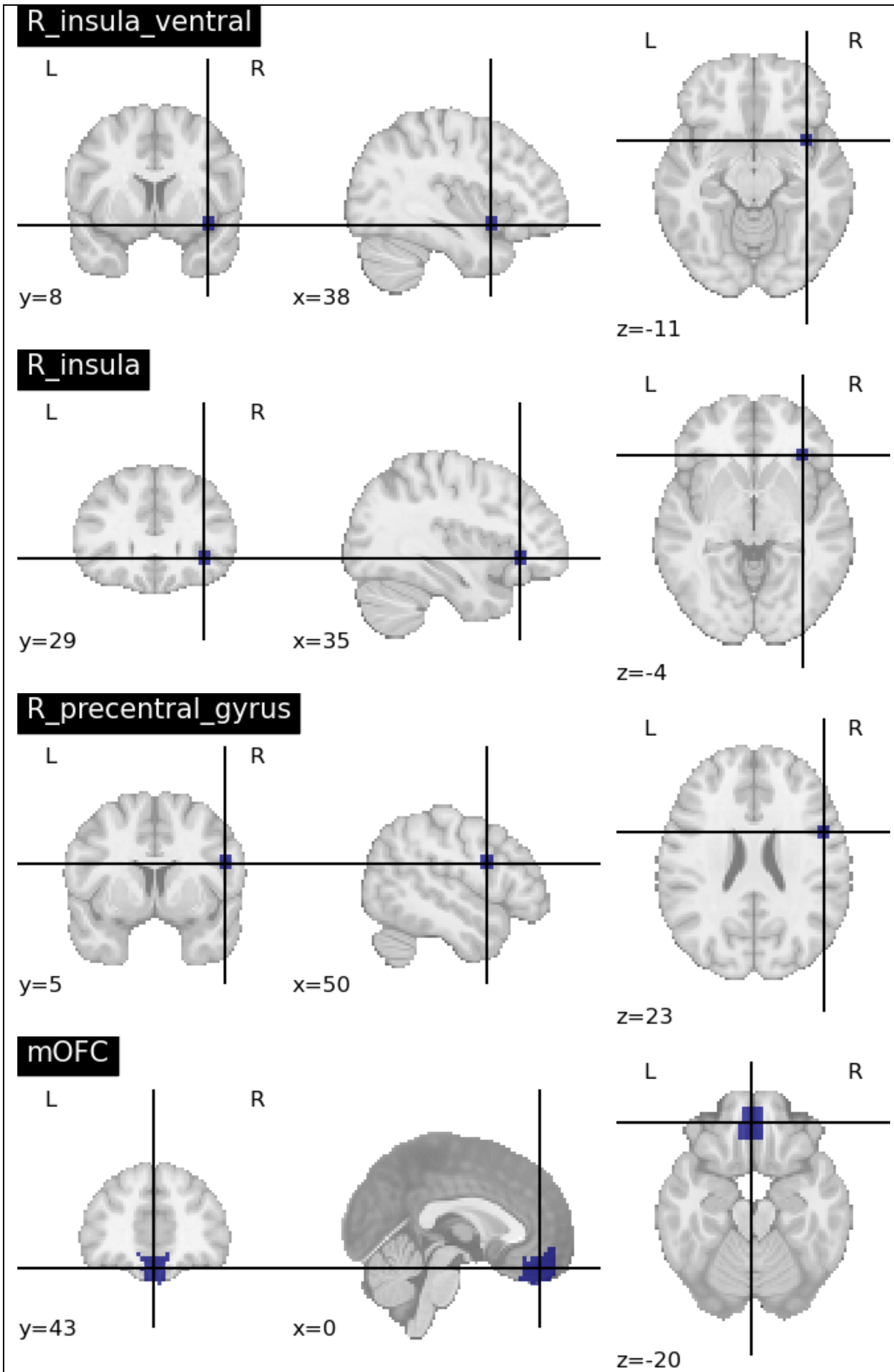

The regions can also be seen and downloaded at https://identifiers.org/neurovault.image:779756.

Supplementary Figure 3: Changes in craving for cigarette smoking from baseline to the 12-week visit based on cigarette smoking status.

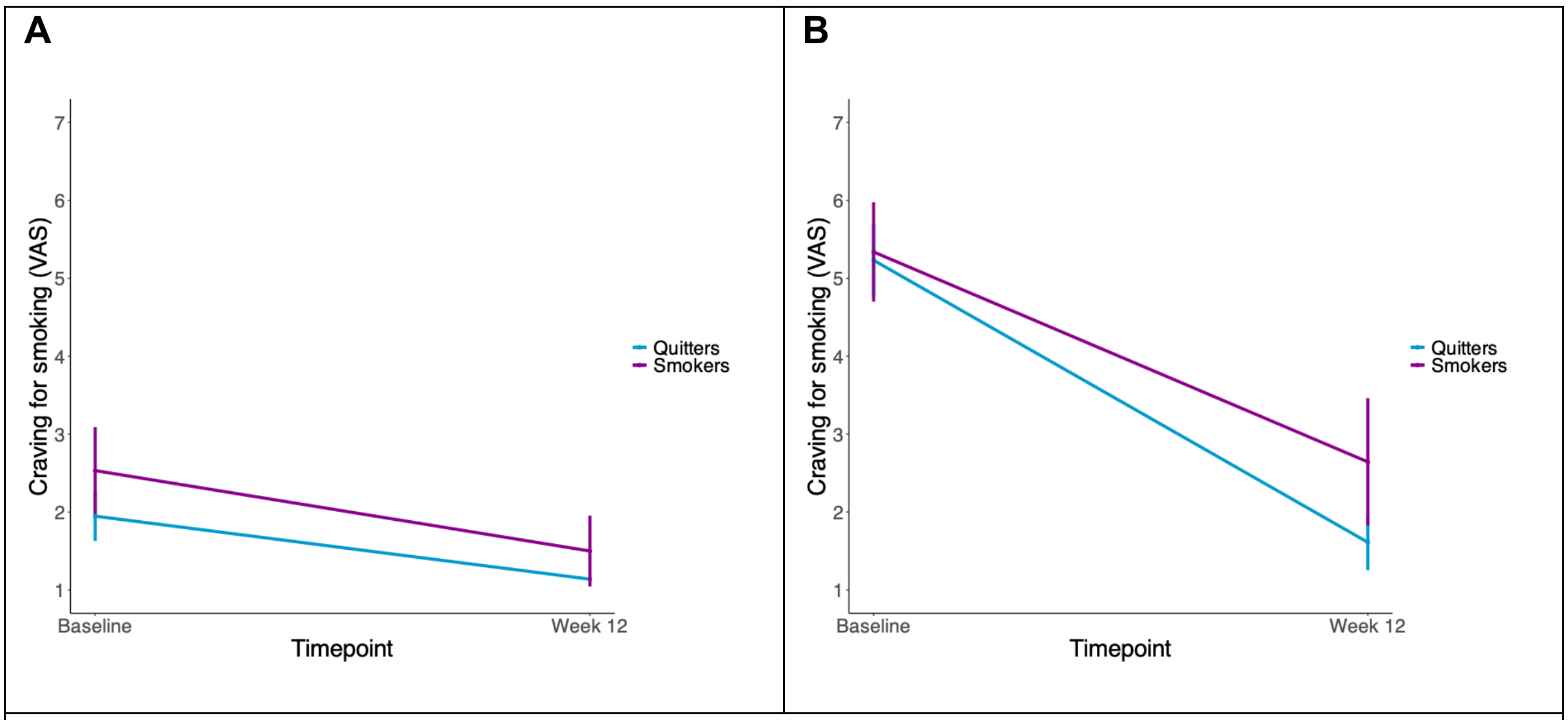

A neutral stimulus only, **B** cigarette smoking stimulus only. Mean craving for cigarette smoking scores (Visual Analogue Scale, VAS) at baseline and Week 12 for quitters (blue line) and persistent smokers (purple line). Data points represent sample mean and error bars indicate the 95% confidence intervals (CI). Total N = 59, Quitters = 36, Persistent smokers = 21.

Supplementary Figure 4: 2-back versus 0-back blocks at baseline, in the whole population.

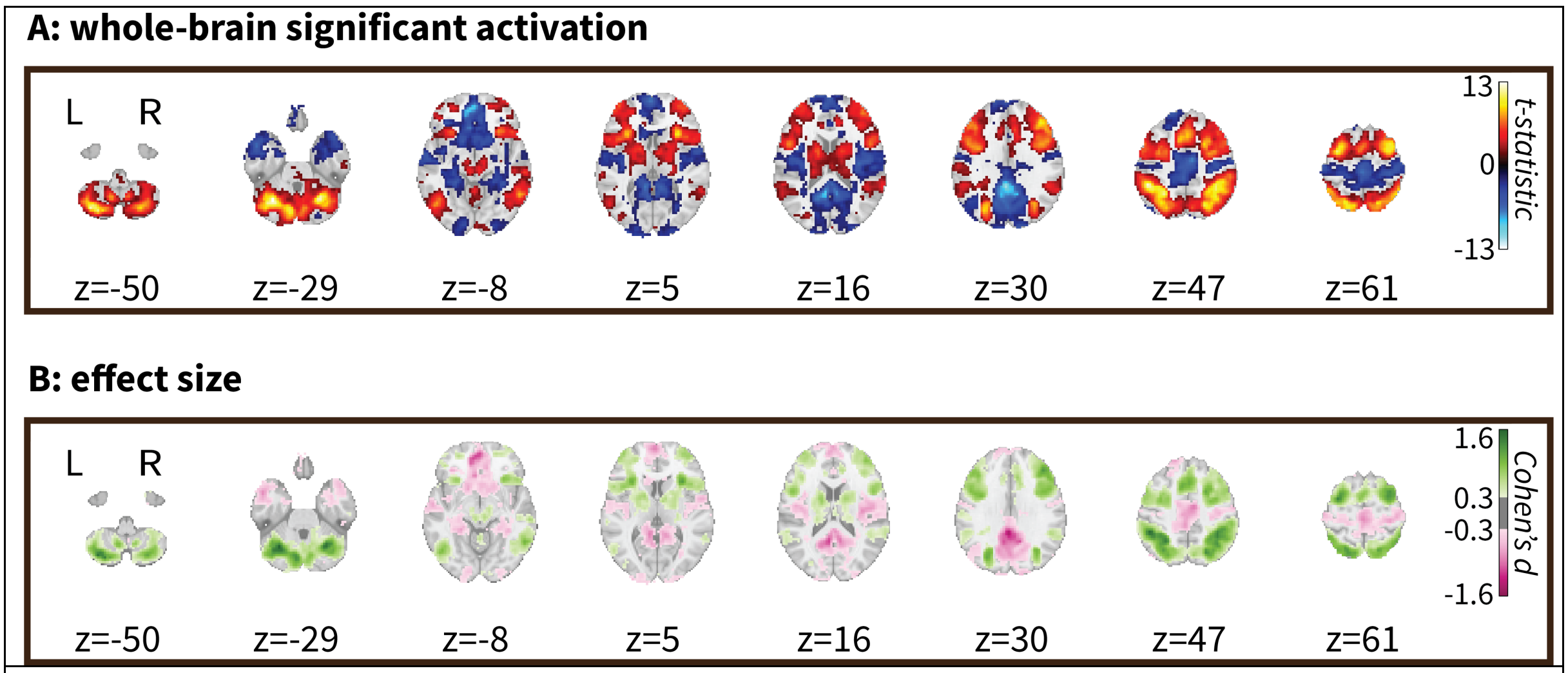

A Whole-brain corrected statistical brain map of the one-sample t-test. Red-yellow colors represent voxels with significantly more activation for 2-back vs 0-back blocks, while dark blue-light blue colors represent significantly more activation for 0-back vs 2-back blocks. The images are corrected for multiple comparisons using familywise error procedure (FWE) (p-corrected <0.05). N = 68.

B Cohen's d effect size map. Green colors represent a greater effect for 2-back vs 0-back blocks, while purple colors represent a greater effect for 0-back vs 2-back blocks. Only values of |d|>0.3 are represented. N = 68.

Z depicts the axial slice's coordinate in MNI (Montreal Neurological Institute) space. L indicates left hemisphere and R indicates right hemisphere.

Supplementary Figure 5: Difference in brain activation for the 2-back versus 0-back blocks between baseline and the 12-week visit, in the whole population.

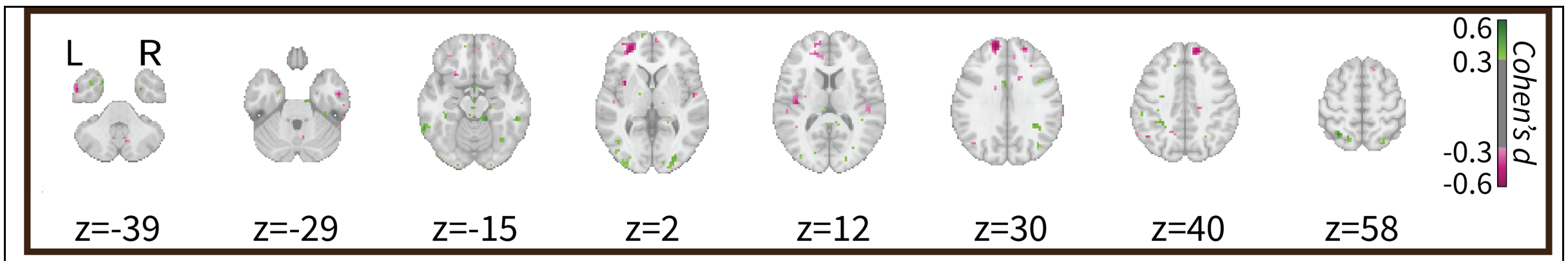

Cohen's d effect size map. Green colors represent an increase in activation from baseline to week12, while purple colors represent a decrease in activation. Only values of |d|>0.3 are represented. N = 54.

Z depicts the axial slice's coordinate in MNI (Montreal Neurological Institute) space. L indicates left hemisphere and R indicates right hemisphere.

Supplementary Figure 6: Resting-state functional connectivity, in the whole population.

A

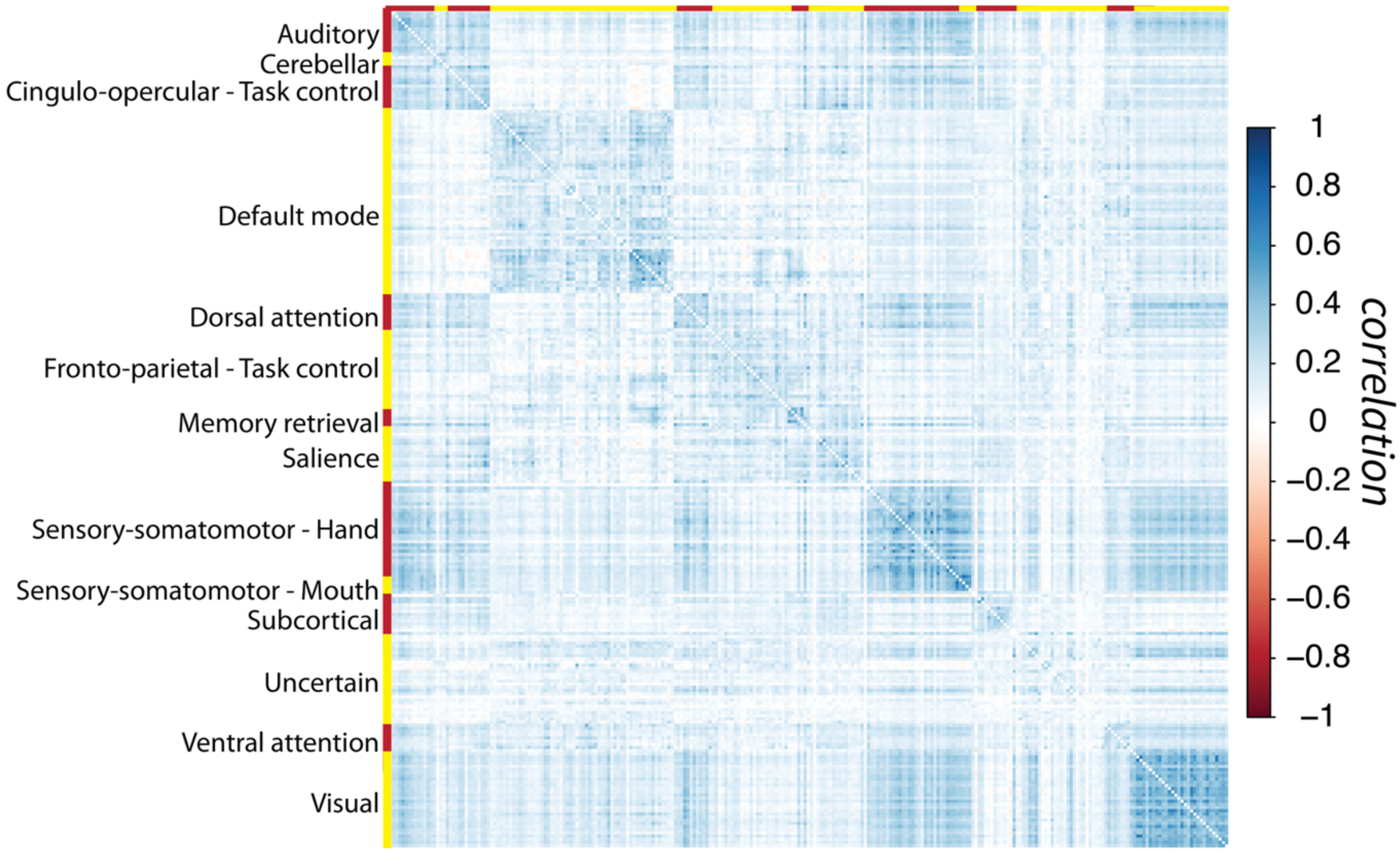

B

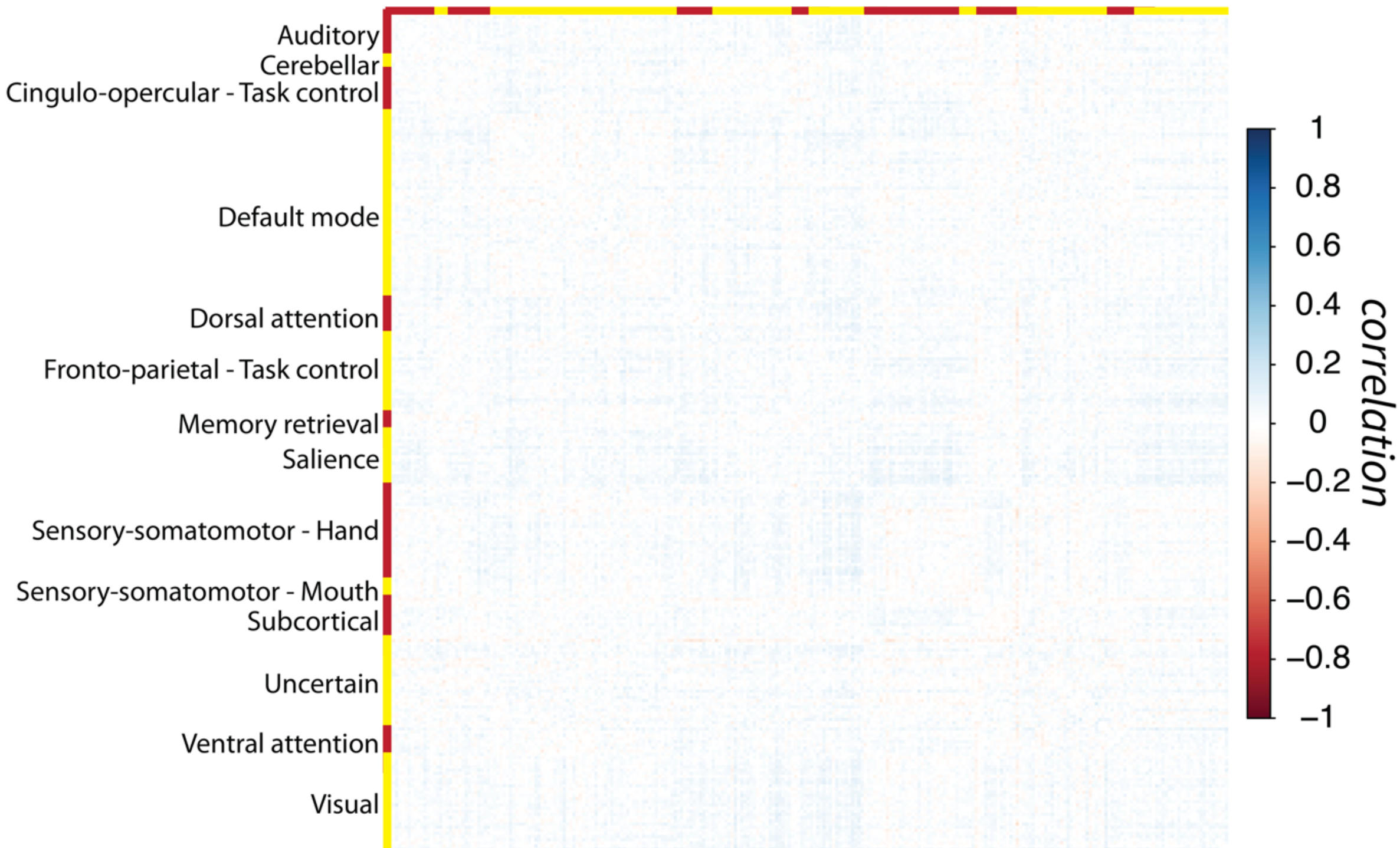

A Baseline (N = 71), **B** Difference between the 12-week visit and baseline (N = 63). Region-to-region functional connectivity is represented as a Pearson's correlation matrix, where the nodes are ordered according to the functional network they belong to.

Supplementary Figure 7: Average perfusion values per region.

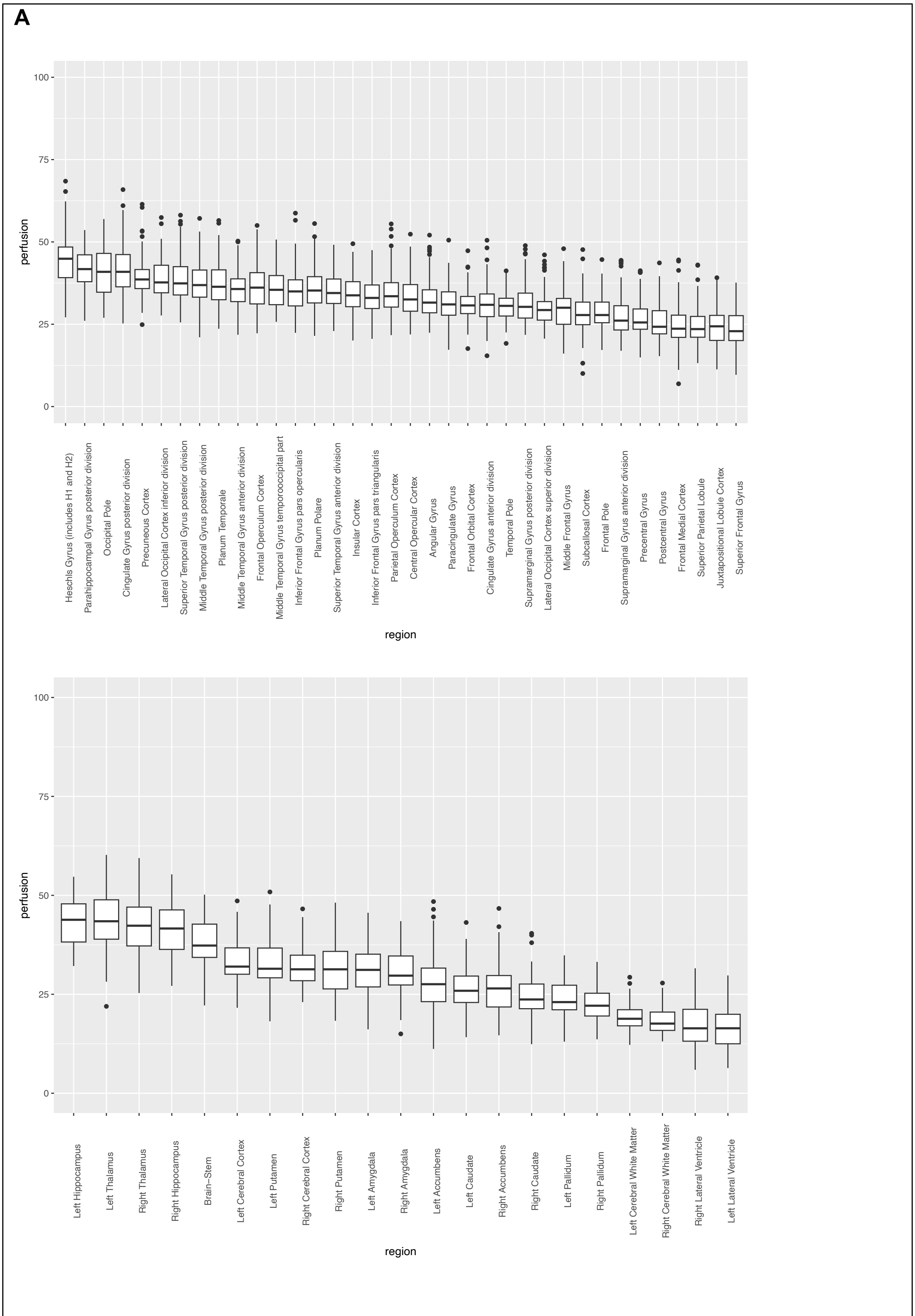

B

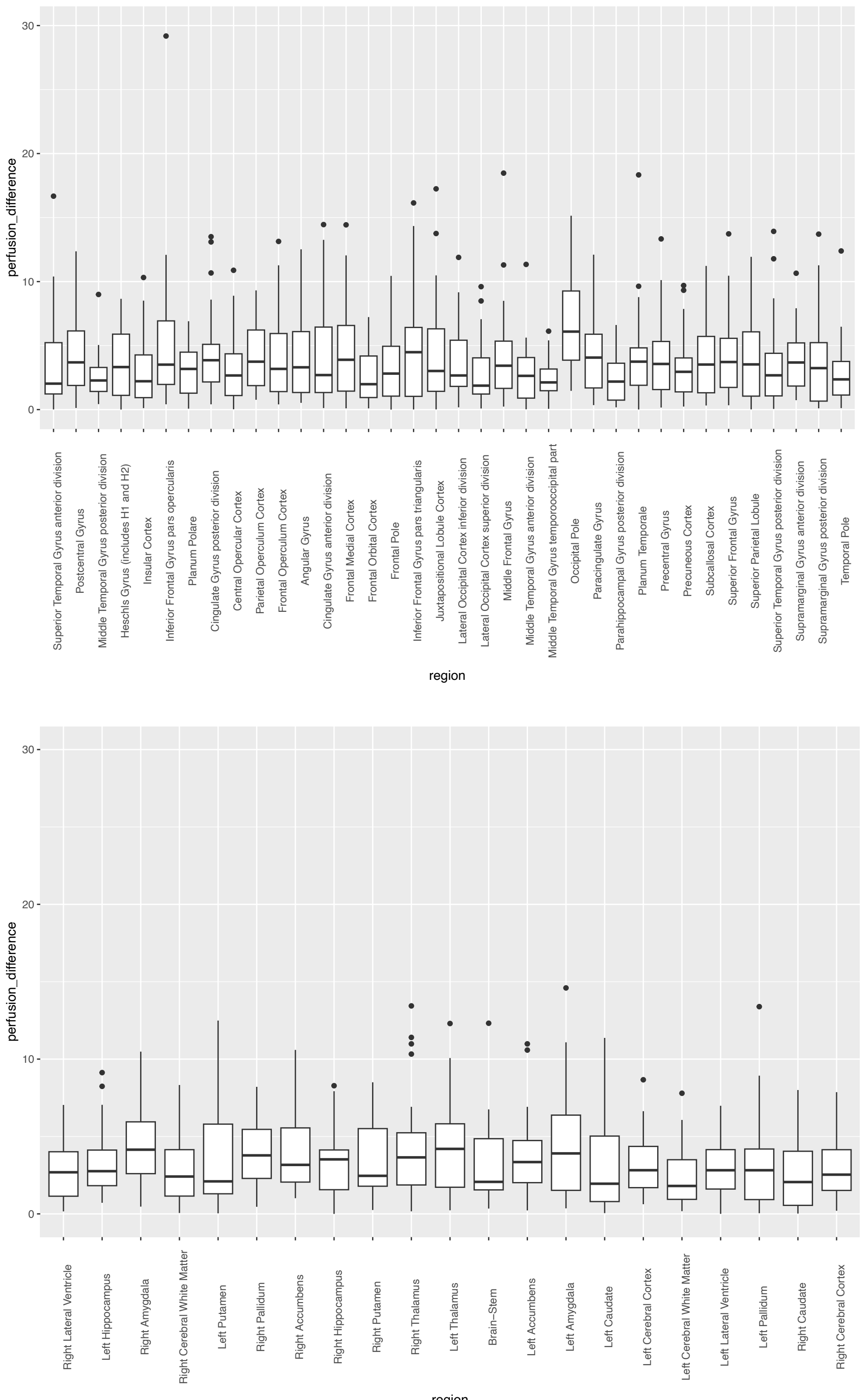

A Baseline perfusion (N = 66); **B** Difference between the 12-week visit and baseline (N = 58). The plots are further divided for cortical and subcortical regions. This figure represents the distribution of the values across participants as boxplots. Individual dots represent subjects for which the perfusion values are outside the +/- 1.5*interquartile range of the group value. Group-level analyses by means of linear models did not reveal any significant differences between sessions (all pFDR>0.05, all t-values>-3.5).

References

1. Heatherton TF, Kozlowski LT, Frecker RC, Fagerström KO. The Fagerstrom Test for Nicotine Dependence: a revision of the Fagerstrom Tolerance Questionnaire. *Addiction*. 1991;86(9):1119-1127. doi:10.1111/j.1360-0443.1991.tb01879.x

2. Müller V, Mucha RF, Ackermann K, Pauli P. Die Erfassung des Cravings bei Rauchern mit einer deutschen Version des “Questionnaire on Smoking Urges“ (QSU-G). *Z Klin Psychol Psychother*. 2001;30(3):164-171. doi:10.1026/0084-5345.30.3.164

3. Jacques Cornuz; Isabelle Jacot Sadowski; Jean-Paul Humair. Tabakprävention. *Ärztliche Rauchstoppberatung - Die Dokumentation für die Praxis Basisdokumentation für Ärzte und Ärztinnen Frei von Tabak*. 2015;(3. Auflage). Accessed December 20, 2021. https://www.fmh.ch/files/pdf20/Basisdokumentation_aerztliche_rauchstoppberatung_D.pdf

4. Cahill K, Lindson-Hawley N, Thomas KH, Fanshawe TR, Lancaster T. Nicotine receptor partial agonists for smoking cessation. *Cochrane Database of Systematic Reviews*. Published online May 9, 2016. doi:10.1002/14651858.CD006103.pub7

5. Brody AL, Mandelkern MA, Olmstead RE, et al. Neural Substrates of Resisting Craving During Cigarette Cue Exposure. *Biol Psychiatry*. 2007;62(6):642-651. doi:10.1016/j.biopsych.2006.10.026

6. Zanchi D, Brody AL, Montandon ML, et al. Cigarette smoking leads to persistent and dose-dependent alterations of brain activity and connectivity in anterior insula and anterior cingulate. *Addiction Biology*. 2015;20(6):1033-1041. doi:10.1111/adb.12292

7. Broome MR, Matthiasson P, Fusar-Poli P, et al. Neural correlates of executive function and working memory in the ‘at-risk mental state.’ *British Journal of Psychiatry*. 2009;194(1):25-33. doi:10.1192/bjp.bp.107.046789

8. Esteban O, Markiewicz CJ, Blair RW, et al. fMRIPrep: a robust preprocessing pipeline for functional MRI. *Nat Methods*. 2019;16(1). doi:10.1038/s41592-018-0235-4

9. Esteban O, Ross Blair CJ, Markiewicz SLB, Craig Moodie F, Ma AII. FMRIPrep. Software. *Zenodo*. Published online 2018. Accessed March 25, 2024. https://doi.org/10.5281/zenodo.852659.

10. Gorgolewski K, Burns CD, Madison C, et al. Nipype: A flexible, lightweight and extensible neuroimaging data processing framework in Python. *Front Neuroinform*. 2011;5. doi:10.3389/fninf.2011.00013

11. Gorgolewski KJ, Oscar Esteban CJ, Markiewicz E, Ziegler DG, Ellis MP, Notter DJ. Nipype. Software. *Zenodo*. Published online 2018. Accessed March 25, 2024. https://doi.org/10.5281/zenodo.596855.

12. Hill-Bowen LD, Riedel MC, Poudel R, et al. The cue-reactivity paradigm: An ensemble of networks driving attention and cognition when viewing drug and natural reward-related stimuli. *Neurosci Biobehav Rev*. 2021;130. doi:10.1016/j.neubiorev.2021.08.010

13. Ciric R, Rosen AFG, Erus G, et al. Mitigating head motion artifact in functional connectivity MRI. *Nat Protoc*. 2018;13(12):2801-2826. doi:10.1038/s41596-018-0065-y

14. Pruim RHR, Mennes M, van Rooij D, Llera A, Buitelaar JK, Beckmann CF. ICA-AROMA: A robust ICA-based strategy for removing motion artifacts from fMRI data. *Neuroimage*. 2015;112:267-277. doi:10.1016/j.neuroimage.2015.02.064

15. Power JD, Cohen AL, Nelson SM, et al. Functional Network Organization of the Human Brain. *Neuron*. 2011;72(4). doi:10.1016/j.neuron.2011.09.006

16. Bates D, Mächler M, Bolker BM, Walker SC. Fitting linear mixed-effects models using lme4. *J Stat Softw*. 2015;67(1). doi:10.18637/jss.v067.i01

17. Jenkinson M, Beckmann CF, Behrens TEJ, Woolrich MW, Smith SM. FSL - Review. *Neuroimage*. 2012;62(2).

18. Makris N, Goldstein JM, Kennedy D, et al. Decreased volume of left and total anterior insular lobule in schizophrenia. *Schizophr Res*. 2006;83(2-3). doi:10.1016/j.schres.2005.11.020

19. Frazier JA, Chiu S, Breeze JL, et al. Structural brain magnetic resonance imaging of limbic and thalamic volumes in pediatric bipolar disorder. *American Journal of Psychiatry*. 2005;162(7). doi:10.1176/appi.ajp.162.7.1256

20. Desikan RS, Ségonne F, Fischl B, et al. An automated labeling system for subdividing the human cerebral cortex on MRI scans into gyral based regions of interest. *Neuroimage*. 2006;31(3). doi:10.1016/j.neuroimage.2006.01.021

21. Goldstein JM, Seidman LJ, Makris N, et al. Hypothalamic Abnormalities in Schizophrenia: Sex Effects and Genetic Vulnerability. *Biol Psychiatry*. 2007;61(8). doi:10.1016/j.biopsych.2006.06.027

22. Reuter M, Schmansky NJ, Rosas HD, Fischl B. Within-subject template estimation for unbiased longitudinal image analysis. *Neuroimage*. 2012;61(4). doi:10.1016/j.neuroimage.2012.02.084

23. Reuter M, Rosas HD, Fischl B. Highly accurate inverse consistent registration: A robust approach. *Neuroimage*. 2010;53(4). doi:10.1016/j.neuroimage.2010.07.020

24. Avants BB, Epstein CL, Grossman M, Gee JC. Symmetric diffeomorphic image registration with cross-correlation: Evaluating automated labeling of elderly and neurodegenerative brain. *Med Image Anal*. 2008;12(1). doi:10.1016/j.media.2007.06.004

25. Tustison NJ, Avants BB, Cook PA, et al. N4ITK: Improved N3 bias correction. *IEEE Trans Med Imaging*. 2010;29(6). doi:10.1109/TMI.2010.2046908

26. Zhang Y, Brady M, Smith S. Segmentation of brain MR images through a hidden Markov random field model and the expectation-maximization algorithm. *IEEE Trans Med Imaging*. 2001;20(1). doi:10.1109/42.906424

27. Klein A, Ghosh SS, Bao FS, et al. Mindboggling morphometry of human brains. *PLoS Comput Biol*. 2017;13(2). doi:10.1371/journal.pcbi.1005350

28. Dale AM, Fischl B, Sereno MI. Cortical surface-based analysis: I. Segmentation and surface reconstruction. *Neuroimage*. 1999;9(2). doi:10.1006/nimg.1998.0395

29. Fonov V, Evans A, McKinstry R, Almli C, Collins D. Unbiased nonlinear average age-appropriate brain templates from birth to adulthood. *Neuroimage*. 2009;47. doi:10.1016/s1053-8119(09)70884-5

30. Glasser MF, Sotiropoulos SN, Wilson JA, et al. The minimal preprocessing pipelines for the Human Connectome Project. *Neuroimage*. 2013;80. doi:10.1016/j.neuroimage.2013.04.127

31. Jenkinson M, Bannister P, Brady M, Smith S. Improved optimization for the robust and accurate linear registration and motion correction of brain images. *Neuroimage*. 2002;17(2). doi:10.1016/S1053-8119(02)91132-8

32. Cox RW, Hyde JS. Software tools for analysis and visualization of fMRI data. *NMR Biomed*. 1997;10(4-5). doi:10.1002/(SICI)1099-1492(199706/08)10:4/5<171::AID-NBM453>3.0.CO;2-L

33. Power JD, Mitra A, Laumann TO, Snyder AZ, Schlaggar BL, Petersen SE. Methods to detect, characterize, and remove motion artifact in resting state fMRI. *Neuroimage*. 2014;84. doi:10.1016/j.neuroimage.2013.08.048

34. Behzadi Y, Restom K, Liau J, Liu TT. A component based noise correction method (CompCor) for BOLD and perfusion based fMRI. *Neuroimage*. 2007;37(1). doi:10.1016/j.neuroimage.2007.04.042

35. Satterthwaite TD, Elliott MA, Gerraty RT, et al. An improved framework for confound regression and filtering for control of motion artifact in the preprocessing of resting-state functional connectivity data. *Neuroimage*. 2013;64(1). doi:10.1016/j.neuroimage.2012.08.052

36. Lanczos C. Evaluation of Noisy Data. *Journal of the Society for Industrial and Applied Mathematics Series B Numerical Analysis*. 1964;1(1). doi:10.1137/0701007

37. Abraham A, Pedregosa F, Eickenberg M, et al. Machine learning for neuroimaging with scikit-learn. *Front Neuroinform*. 2014;8(FEB). doi:10.3389/fninf.2014.00014
